# Antibiotic-induced release of small extracellular vesicles (exosomes) with surface-associated DNA

**DOI:** 10.1038/s41598-017-08392-1

**Published:** 2017-08-15

**Authors:** Andrea Németh, Norbert Orgovan, Barbara W Sódar, Xabier Osteikoetxea, Krisztina Pálóczi, Katalin É. Szabó-Taylor, Krisztina V. Vukman, Ágnes Kittel, Lilla Turiák, Zoltán Wiener, Sára Tóth, László Drahos, Károly Vékey, Robert Horvath, Edit I. Buzás

**Affiliations:** 1 Department of Genetics, Cell- and Immunobiology, Semmelweis University, Budapest, 1085 Hungary; 2 Institute of Technical Physics and Materials Science, Hungarian Academy of Sciences, Budapest, 1121 Hungary; 3 Institute of Experimental Medicine, Hungarian Academy of Sciences, Budapest, 1083 Hungary; 4 Research Centre for Natural Sciences, Hungarian Academy of Sciences, Budapest, 1117 Hungary

## Abstract

Recently, biological roles of extracellular vesicles (which include among others exosomes, microvesicles and apoptotic bodies) have attracted substantial attention in various fields of biomedicine. Here we investigated the impact of sustained exposure of cells to the fluoroquinolone antibiotic ciprofloxacin on the released extracellular vesicles. Ciprofloxacin is widely used in humans against bacterial infections as well as in cell cultures against *Mycoplasma* contamination. However, ciprofloxacin is an inducer of oxidative stress and mitochondrial dysfunction of mammalian cells. Unexpectedly, here we found that ciprofloxacin induced the release of both DNA (mitochondrial and chromosomal sequences) and DNA-binding proteins on the exofacial surfaces of small extracellular vesicles referred to in this paper as exosomes. Furthermore, a label-free optical biosensor analysis revealed DNA-dependent binding of exosomes to fibronectin. DNA release on the surface of exosomes was not affected any further by cellular activation or apoptosis induction. Our results reveal for the first time that prolonged low-dose ciprofloxacin exposure leads to the release of DNA associated with the external surface of exosomes.

## Introduction

Extracellular vesicles (EVs) play key roles in intercellular communication by which they may impact a wide range of biological functions of cells. EVs are phospholipid bilayer enclosed particles that can deliver lipids, proteins, nucleic acids, carbohydrates and metabolites to both neighboring and distant cells^1, 2^. EVs are heterogeneous in their biogenesis, molecular composition and size^2–4^.

Exosomes (EXOs) are released from cells during exocytosis of multivesicular bodies into the extracellular space^1, 2, 5, 6^. EXOs typically represent the smallest sized (~100 nm) EVs. Microvesicles (MVs) alternatively designated as microparticles or shedding vesicles or ectosomes, are usually intermediate-sized vesicles (~100–1000 nm). They shed from the cell surface by outward budding of the plasma membrane^1, 2, 5, 6^. Large vesicles with diameter >1 µm can be produced during apoptosis (in which case they are referred to as apoptotic bodies, APOs)^1, 4, 5^. Of note, highly migratory tumor cells also release large vesicles (referred to as large oncosomes) of several µm in diameter^7^. Although there might be exceptions, the above size range categories apply for the vast majority of EVs of endosomal or plasma membrane origin. Even if the biogenesis of these EV subpopulations was not investigated specifically in this study, we decided to use the terms EXO, MV and APO for EVs in the above size categories.

EVs can alter signaling of recipient cells by either cell surface receptor-ligand interactions or upon uptake by cells. EVs have been shown to deliver specific mRNAs and various small RNAs^8–10^ as well as DNA^11–15^ to healthy cells. They modify the genetic composition of recipient cells and alter their functions^12, 16–19^. EXOs have been shown to carry DNase-resistant intravesicular DNA, protected by a phospholipid bilayer membrane. The mutation status of this DNA was comparable to that of the cell of origin^13, 15, 20^. Moreover, studies also showed that cells release EXOs containing mitochondrial DNA (mtDNA)^21, 22^. Until now, most studies focused exclusively on intraexosomal DNA, and DNase digestion was mainly used to eliminate any potential contaminating extravesicular DNA^15, 23, 24^. As far as the potential external association of DNA with the exosomal surface is concerned, Cai *et al*. found no DNase I-sensitive external DNA in human plasma- and vascular smooth muscle cell-derived exosomal samples^12^. In contrast, in a recent short report, a human mast cell line was shown to carry DNAse I-sensitive nucleic acid on the surface of EXOs^25^. In the same study, it was suggested that this DNA might play a role in the cellular uptake of EXOs^25^. Moreover, Fisher *et al*. found external, DNase I-sensitive nuclear DNA in EV samples released by mesenchymal stem cells^17^. In yet another publication DNAse sensitive chromatin was shown on the surface of EVs secreted into blood^26^. This EV-associated chromatin as a potential self-antigen could induce an anti-DNA antibody response. The study suggested that as a defense mechanism, dendritic cells and macrophages produced extracellular DNase for digestion of EV-associated chromatin, and thus, inhibited autoimmune reactions^26^.

In the present study, we investigated, the effects of the fluoroquinolone antibiotic ciprofloxacin that is used in the therapy of several human bacterial infections and is also applied *in vitro* against *Mycoplasma* contamination of cell cultures. The presence of a clinically relevant dose of ciprofloxacin has been reported to cause oxidative damage, mitochondrial dysfunction and mtDNA depletion in mammalian cells^27–29^. Here we report for the first time that ciprofloxacin induced the release of both mitochondrial and chromosomal DNA associated with the surface of EXOs. We also demonstrate that this exofacial DNA facilitates EXO binding to the extracellular matrix protein fibronectin.

## Results

### Sustained exposure of cells to ciprofloxacin induces the release of DNA associated with EVs

**Figure 1:**
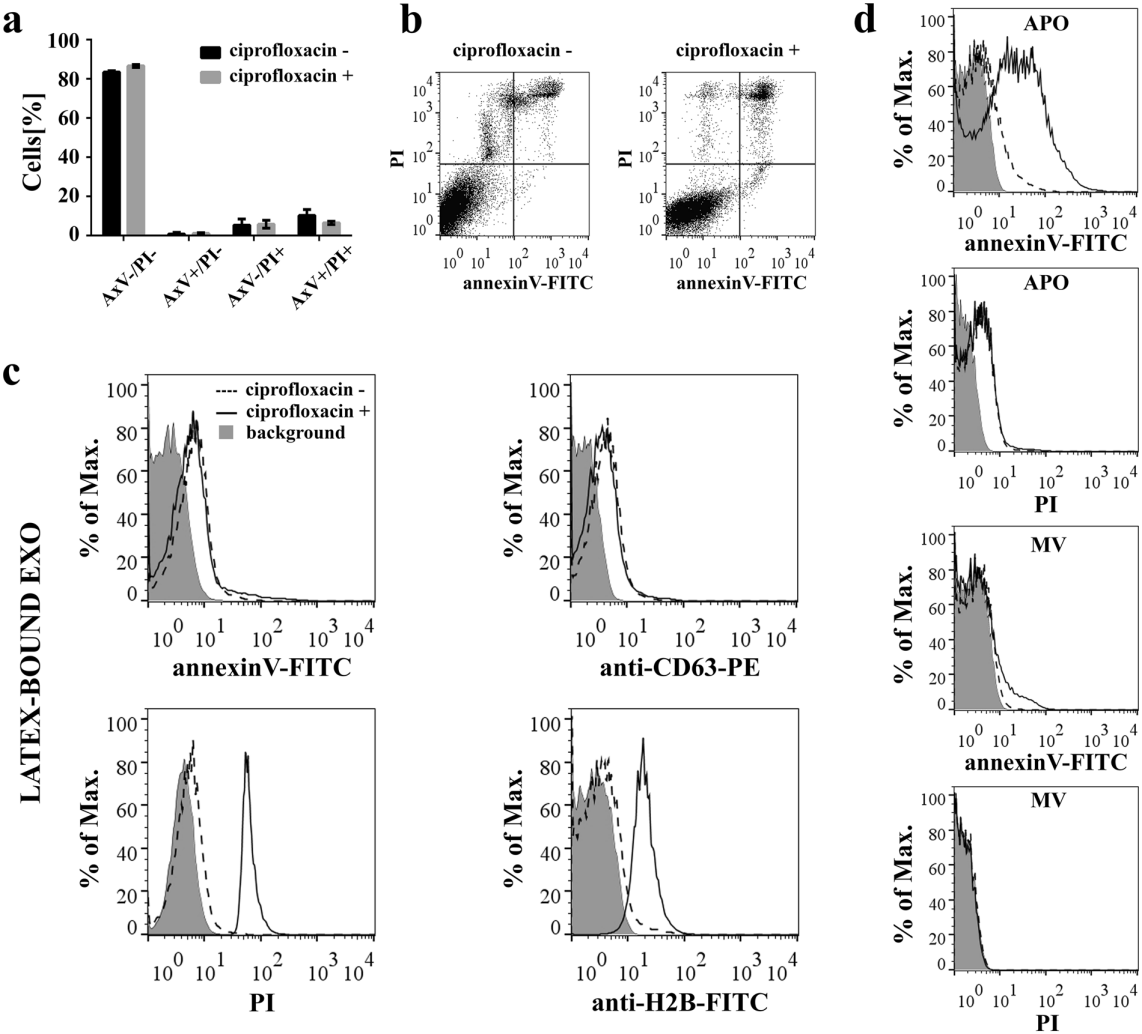
Effects of sustained ciprofloxacin exposure on Jurkat cells. **(a,b)** Viability of Jurkat cells with/without exposure to ciprofloxacin (10 µg/mL for >14 days) was analyzed by flow cytometry after staining with annexinV-FITC and propidium iodide (PI). **(a)** Mean values+/− S.D. (error bars) of two independent experiments are shown in the histogram plot. AxV: annexinV. **(b)** Representative dot plots showing the four quadrants of annexinV-FITC and PI stained Jurkat cells. **(c)** Exosomes (EXOs) derived from ciprofloxacin-exposed/unexposed Jurkat cells were conjugated onto latex beads and characterized by flow cytometry after staining with annexinV-FITC, anti-CD63-PE, PI or anti-histone H2B-FITC. The background fluorescence of stained latex beads is indicated by grey histograms. **(d)** Microvesicles (MVs) and apoptotic bodies (APOs) were labeled directly with annexinV-FITC and PI. The background fluorescence of EVs is indicated by grey histograms.

We first compared Jurkat cells with or without a sustained (>14 days) exposure to ciprofloxacin. In line with previous observations by others^27, 30^, we found that the presence of this low-dose (10 µg/mL) antibiotic did not have a significant effect on cell viability (Fig. 1a and b). Moreover, also in agreement with previous published findings^27–29, 31^, our mass spectrometry (MS) analysis of cells showed that the presence of ciprofloxacin resulted in a slightly elevated percentage of cellular proteins associated for example with oxidative stress and defense responses, mitochondrial degradation, and in a somewhat reduced percentage of respiratory electron transport chain-associated proteins (Supplementary Fig. S1, Supplementary Dataset S1). Of note, all the observed minute proteomic differences were in line with previously published data^27^, and were found reproducibly in two independent experiments.

Strikingly, when analyzing size-based fractions of EVs secreted by Jurkat cells, flow cytometry revealed an unexpected difference between EXOs released in the presence or absence of ciprofloxacin. We found that ciprofloxacin-exposed Jurkat cells (but not those cultured without this antibiotic) released EXOs with substantial propidium iodide (PI) and anti-histone H2B staining (Fig. 1c). Given that we found comparable annexinV and CD63 positivities both in the presence and absence of ciprofloxacin (Fig. 1c), we concluded that the differences in PI and anti-H2B stainings were not due to differences in the amounts of the released EXOs. Moreover, APOs and to a much lesser extent, MVs, showed higher annexinV positivity in the presence of ciprofloxacin (Fig. 1d). In contrast, there was no difference in the PI staining of APOs or MVs released by ciprofloxacin-exposed and unexposed cells (Fig. 1d). APOs and MVs were found negative for histone H2B (Supplementary Fig. S2).

In order to study if the observed effect of ciprofloxacin was specific to Jurkat cells, EVs were isolated from the MiaPaCa human pancreatic cancer cell line and from the U937 human monocytic cells, and were analyzed by flow cytometry. Our results show that the presence of ciprofloxacin induced a robust EXO-associated DNA secretion by MiaPaCa cells (Supplementary Fig. S3). However, we did not observe this phenomenon in the case of U937 cells (Supplementary Fig. S3).

### Assessment of ciprofloxacin-induced DNA in size-based EV fractions of Jurkat cells

**Figure 2:**
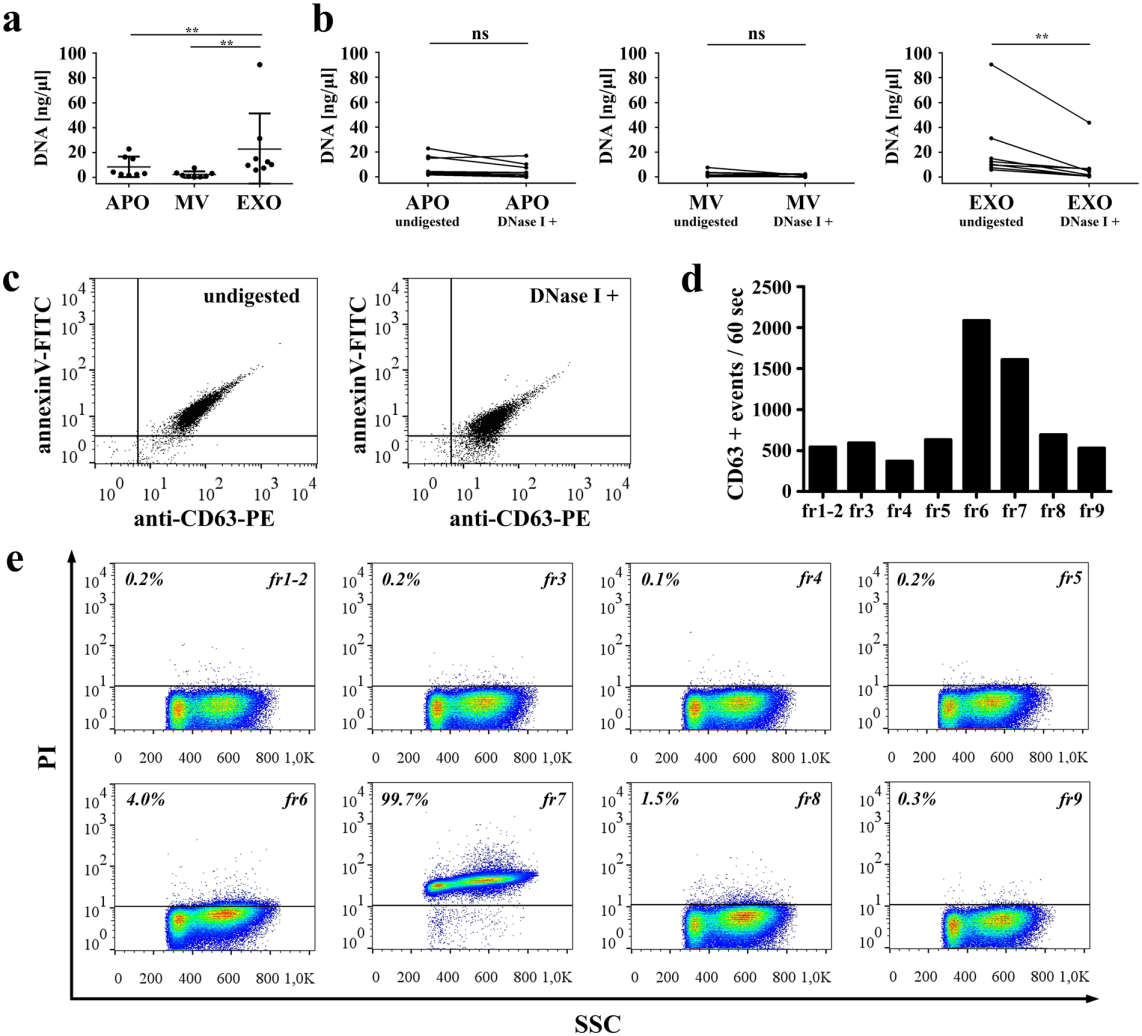
Analysis of ciprofloxacin-induced release of DNA associated with extracellular vesicles (EVs). **(a)** DNA content of size-based EV fractions determined with/without DNase I digestion of EVs. Concentration values represent the amount of EV-associated DNA (eluted in 3 0 µL) isolated from the conditioned medium of 2.5 × 10^7^ Jurkat cells. Plotted values are presented as the mean+/− S.D. (error bars) of 8 independent experiments. (**P < 0.01, Friedman test, One-way ANOVA). **(b)** DNA amounts before and after the digestion of EVs by DNase I. The paired measurements (n = 8) for APOs, MVs and EXOs are indicated by lines (**P < 0.01, Wilcoxon signed rank test). APO: apoptotic body, MV: microvesicle, EXO: exosome **(c)** The presence of EXOs both in undigested and DNase I digested samples was confirmed by flow cytometry. Latex-bound EXOs were stained with annexinV-FITC and an anti-CD63-PE antibody. Dot plots are representative of three independent experiments. **(d)** Optiprep^TM^ density gradient fractions of the 100,000 g pellet (containing EXOs) were re-pelleted, conjugated onto latex beads and stained with an anti-CD63-PE antibody for flow cytometry. **(e)** The same latex-bound Optiprep^TM^ density gradient fractions were also stained by propidium iodide (PI). The percentages of PI positive events are shown above a threshold (horizontal line, determined by measuring labeled latex beads without conjugated density gradient fractions).

Next, we compared the ciprofloxacin-induced DNA content of the Jurkat cell-derived EV samples. We found that all EV fractions carried DNA. However, DNA was mainly associated with the EV fraction containing EXOs (Fig. 2a, **P < 0.01, Friedman test, One-way ANOVA). Furthermore, the amount of EXO fraction-associated DNA decreased significantly upon digestion of EXOs with DNAse I (Fig. 2b, **P < 0.01, Wilcoxon signed rank test). By staining DNase I-digested and undigested EXO samples with annexinV and anti-CD63, we ruled out the possibility that the reduced DNA staining after DNase I digestion was due to the loss of EXOs (Fig. 2c). These data suggest that a substantial portion of the EXO fraction-associated DNA was not protected by the phospholipid bilayer membrane of EXOs.

To confirm that the DNA in the 100,000 g EXO pellet was associated with the surface of EXOs (rather than being inside of them), we ran our samples on an Optiprep^TM^ density gradient. Collected fractions were re-pelleted, and analyzed by flow cytometry. Using an anti-CD63 antibody we confirmed that EXOs floated both in fractions 6 and 7 (at densities 1.07 and 1.09 g/mL, respectively) (Fig. 2d). Strikingly, PI staining was only detectable in fraction 7 (Fig. 2e). This suggests that DNA was found at 1.09 g/mL density together with EXOs but not with EXOs that floated at 1.07 mg/mL density (fraction 6) (Fig. 2e). Densities of Optiprep^TM^ fractions are listed in Supplementary Table S1. In Optiprep^TM^, the buoyant density of DNA is around 1.20–1.22 g/mL^32^, while EXOs have a lower buoyant density^33^. Thus, the co-localization within the same density gradient fraction may suggest the association of DNA and EXOs.

**Figure 3:**
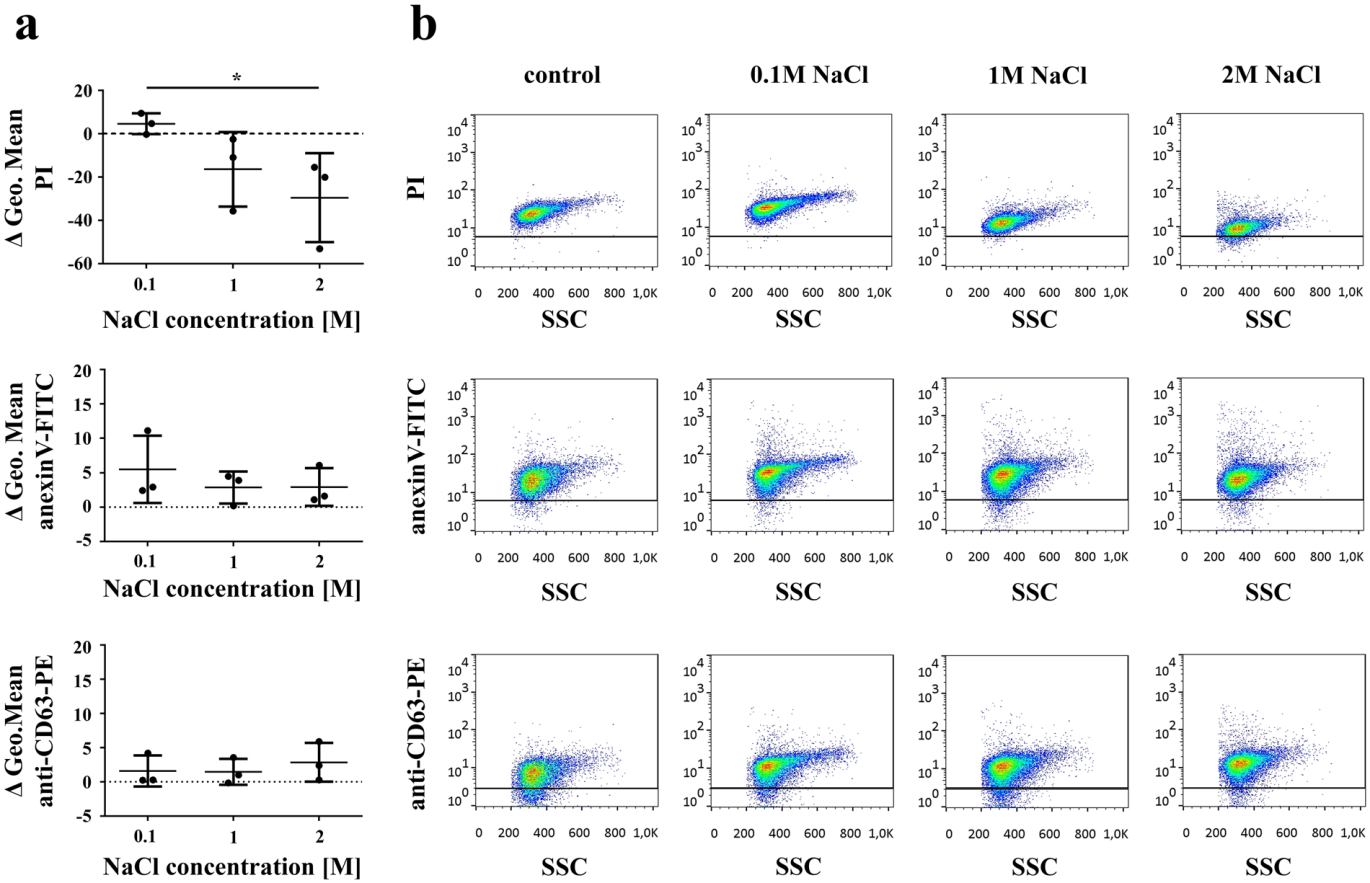
Association of DNA with EXO surface is abolished by high salt concentration. Ciprofloxacin-exposed Jurkat cell exosomes (EXOs) were conjugated onto latex beads and washed using annexinV binding buffer supplemented with 0.1 M, 1 M or 2 M NaCl. All latex-bound EXO samples were re-suspended in annexinV binding buffer, and labeled with propidium iodide (PI), annexinV-FITC and an anti-CD63-PE antibody for flow cytometry. **(a)** Differences of geometric mean fluorescence values between EXOs washed in high salt concentration buffers and controls are shown. Plotted values are presented as the mean+/− S.D. (error bars) of three independent experiments. *P < 0.05, Friedman test, One-way ANOVA **(b)** Density plots show PI, annexinV-FITC and anti-CD63-PE fluorescence of latex-bound EXOs as a function of SSC parameter. Horizontal lines indicate fluorescence threshold of labeled EXO-free latex beads.

Next, we tested if the EXO-associated DNA could be eluted from the surface of EXOs in the presence of high salt concentration. We found that washing latex-bound EXOs in 2 M NaCl resulted in a significant decrease (Fig. 3, *P < 0.05, Friedman test, One-way ANOVA) in the PI fluorescence intensity of stained EXOs. In contrast, high concentration of NaCl did not lead to a decrease in either CD63 or annexinV positivities (Fig. 3). This indicates that the high salt concentration released DNA from EXOs rather than detaching EXOs from the beads. PI positivity of latex-bound DNA (without the presence of EXOs) was low, and was not reduced under the above conditions, suggesting that DNA was indeed released from the EXO surfaces rather than from the latex beads (Supplementary Fig. S4). Background staining of latex beads without any attached EXOs or purified DNA was also assessed by flow cytometry (Supplementary Fig. S5).

### Surface binding of EVs with exofacial DNA

**Figure 4:**
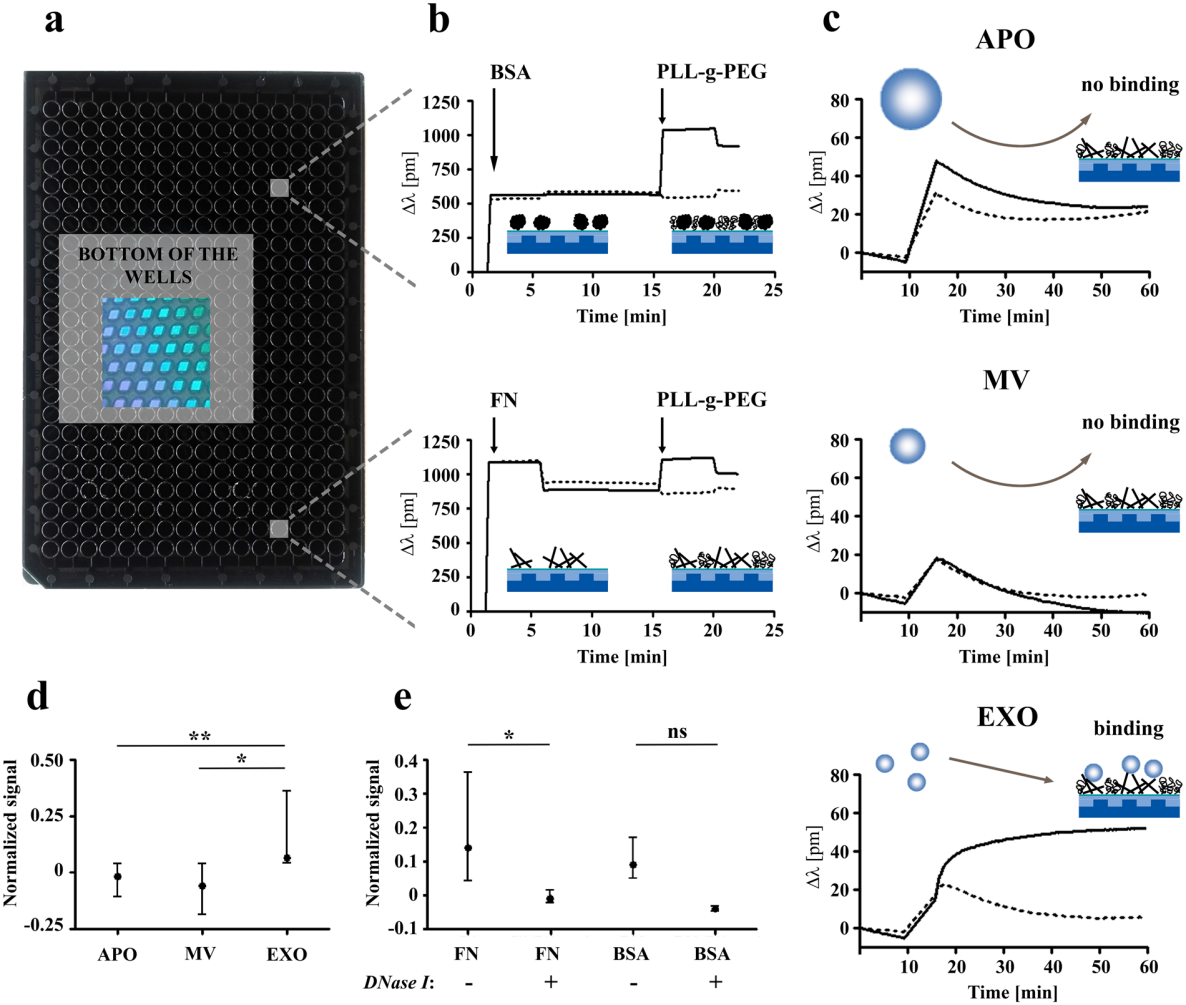
Label-free optical biosensor analysis of surface adhesion of extracellular vesicles (EVs). **(a)** Photograph of an Epic microplate (384-well) is shown containing biosensors (2 × 2 mm nano-grating embedded in a high-refractive index waveguiding film) at the bottom of each well. Biosensors are imaged from the back of the plate and are visible due to diffraction. **(b)** Microplate wells were coated with bovine serum albumin (BSA) as a control protein or with fibronectin (FN) resulting in a shift in the resonant wavelength (Δλ). Microplate wells were equilibrated with PBS, then BSA or FN were added to the wells (indicated by the first arrows). After one hour incubation with BSA or FN, Δλ was recorded for 5 min. Then, unbound protein was washed out with PBS, and Δλ was measured again for 10 min. Then, PLL-g-PEG was used in order to block the non-specific binding sites of wells. The BSA and FN adsorption signals without addition of PLL-g-PEG (PBS only) are indicated as dashed lines, while adsorption of the blocking PLL-g-PEG in BSA- or FN-coated wells is shown by continuous line. After 30 min incubation with PLL-g-PEG, Δλ was recorded for another 5 min (starting points are indicated by the second arrows), and finally PLL-g-PEG was changed to PBS. **(c)** Adsorption of apoptotic bodies (APOs), microvesicles (MVs) and exosomes (EXOs) onto FN + PLL-g-PEG surfaces (continuous lines) or onto surfaces with adsorbed PLL-g-PEG only (dashed lines). **(d)** The Δλ values of EV adsorption onto PLL-g-PEG were subtracted from adsorption values onto FN + PLL-g-PEG, and were divided by the Δλ value of EV adsorption onto bare surfaces (as a straightforward normalization with the mass concentrations of various samples). These normalized signal values are presented as the mean+/− S.D. (error bars) of three independent experiments (**P < 0.01 between APOs and EXOs, *P < 0.05 between MVs and EXOs, Mann-Whitney U-test). **(e)** Comparison of EXO adsorption with/without DNase I digestion onto FN + PLL-g-PEG and onto BSA + PLL-g-PEG surfaces. Normalized signal values are presented as the mean+/− S.D. (error bars) of three independent experiments. Difference was significant in the case of FN surface (*P < 0.05, Mann Whitney U-test). PLL-g-PEG: poly(L-lysine)-*graft*-poly(ethylene glycol).

Thereafter, we asked if the exosomal surface-associated DNA had an effect on the adhesion properties of EXOs. We tested the adherence of size-based EV fractions using a resonant waveguide grating-based label-free optical biosensor (Fig. 4a). Adsorption of fibronectin (FN) or bovine serum albumin (BSA) as a control protein onto the bottom of the biosensor wells resulted in large signals, which did not decrease after the PBS washing step. This suggests that these biomolecules irreversibly adsorbed onto the niobium-pentoxide (Nb_2_O_5_) surface (Fig. 4b). Importantly, the ciprofloxacin-exposed Jurkat cell-derived APOs, MVs, and EXOs adsorbed onto the bare biosensor surface nonspecifically, and the resulting signals were proportional with the concentration of the samples. In order to exclude mass concentration differences of EVs, we used normalized adsorption signal values by taking into account the signals measured using the bare biosensor surfaces. APOs and MVs did not bind to FN coating (Figs. 4c and d). In sharp contrast, EXOs adhered significantly (Fig. 4d, **P < 0.01 between APOs and EXOs, *P < 0.05 between MVs and EXOs, Mann-Whitney U-test) onto the FN-coated surface of the wells. Next, we tested the possibility that exofacial DNA on EXOs could play a role in this EXO-FN interaction. Figure 4e shows that adhesion of EXOs onto FN was reduced significantly (*P < 0.05, Mann Whitney U-test) upon DNase I digestion. DNase I solution alone resulted in negligible contribution to the results (Supplementary Fig. S6).

### Activation or apoptosis induction of ciprofloxacin-exposed Jurkat cells

We also wondered whether activation or apoptosis induction had an impact on the association of DNA with EVs of ciprofloxacin-exposed Jurkat cells. Conditions for cellular activation and apoptosis induction were selected by pilot experiments (Supplementary Figures S7-S9). Activation and apoptosis of cells were confirmed by nonyl-acridin orange (NAO), annexinV, PI, PKH67 and DAPI stainings (Supplementary Fig. S10).

**Figure 5:**
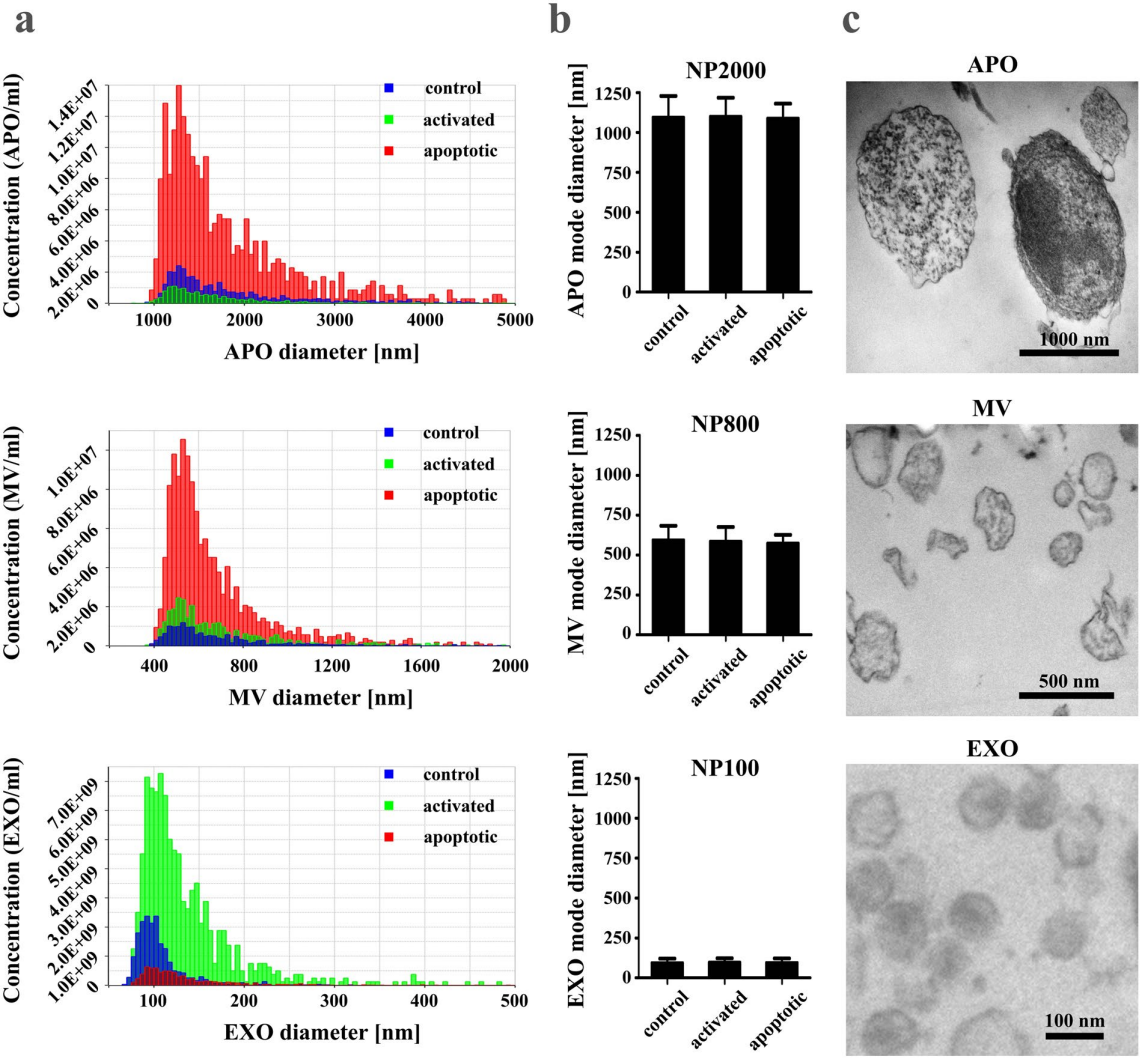
Characterization of extracellular vesicles (EVs) released by ciprofloxacin-exposed control, activated or apoptotic Jurkat cells. (**a**) Concentration values of EVs in 100 µL (isolated from the supernatant of 2.5 × 10^7^ cells) were determined by tunable resistive pulse sensing (TRPS), and are plotted as a function of their diameter for control, activated or apoptotic cells (blue, green or red colors, respectively). Histogram plots of EVs are representative of three independent experiments. (**b**) Mode diameters of EV subpopulations were determined by TRPS, and are presented+/− S.D. (error bars) of three independent experiments, for control, activated and apoptotic cells. NP2000, NP800 and NP100 IZON nanopore membranes were used. (**c**) Transmission electron microscopy images of apoptotic bodies (APOs), microvesicles (MVs) and exosomes (EXOs) samples.

Next we set out to analyze the secreted EVs with tunable resistive pulse sensing. Figure 5a shows that apoptotic Jurkat cells secreted higher number of APOs and MVs than either the controls or the activated cells. In contrast, a very high number of EXOs (two orders of magnitude higher than APOs and MVs) was secreted by activated cells (Fig. 5a). The mode diameter of the secreted vesicles did not differ significantly in the different functional states of the producing cells (Fig. 5b). Transmission electron microscopy confirmed the presence of the respective vesicles (APOs, MVs and EXOs) in the size-based EV fractions (Fig. 5c).

**Figure 6:**
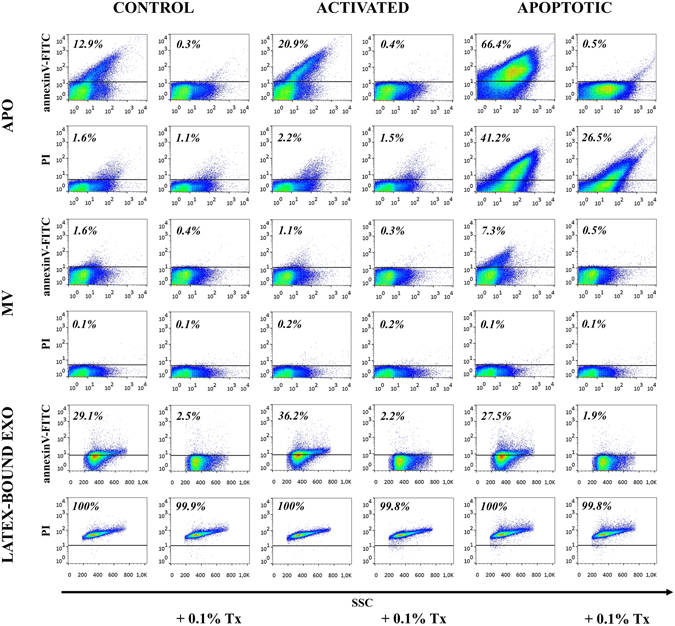
Flow cytometry analysis of extracellular vesicles (EVs) derived from ciprofloxacin-exposed control, activated or apoptotic Jurkat cells. EVs were stained by annexinV-FITC and propidium iodide (PI) for flow cytometry and analyzed before and after detergent lysis with 0.1% Triton X-100. Apoptotic bodies (APOs) and microvesicles (MVs) were labeled and measured directly, whereas exosomes (EXOs) were conjugated onto latex beads before staining. Density plots show annexinV-FITC and PI positivity of EVs derived from ciprofloxacin-exposed control, activated and apoptotic Jurkat cells. The percentages of positive events (above the threshold represented by a black line) are shown in the plots.

Thereafter, we characterized EVs by flow cytometry (Fig. 6). All APOs and MVs derived from control, activated, and apoptotic Jurkat cells showed annexinV positivity, which was particularly strong in the case of APOs. AnnexinV staining diminished after lysis with 0.1% Triton X-100 indicating the presence of detergent-sensitive membrane-enclosed vesicular structures^34^. APOs were positively stained with PI, and the number of PI positive events in the APO gate showed a robust increase upon apoptosis induction. However, in contrast to annexinV positivity, PI staining of APOs was not abolished by 0.1% Triton X-100, suggesting that molecules within these large vesicles were possibly cross-linked by transglutaminases and were resistant to detergent lysis. All MVs were negative for PI (Fig. 6). Staining of latex-bound EXOs with annexinV confirmed the presence of externalized phophatidylserine, and was also substantially reduced by the 0.1% Triton X-100 lysis. PI staining of EXOs indicated the presence of DNA in all EXO samples and was insensitive to Triton X-100 treatment (Fig. 6).

**Figure 7:**
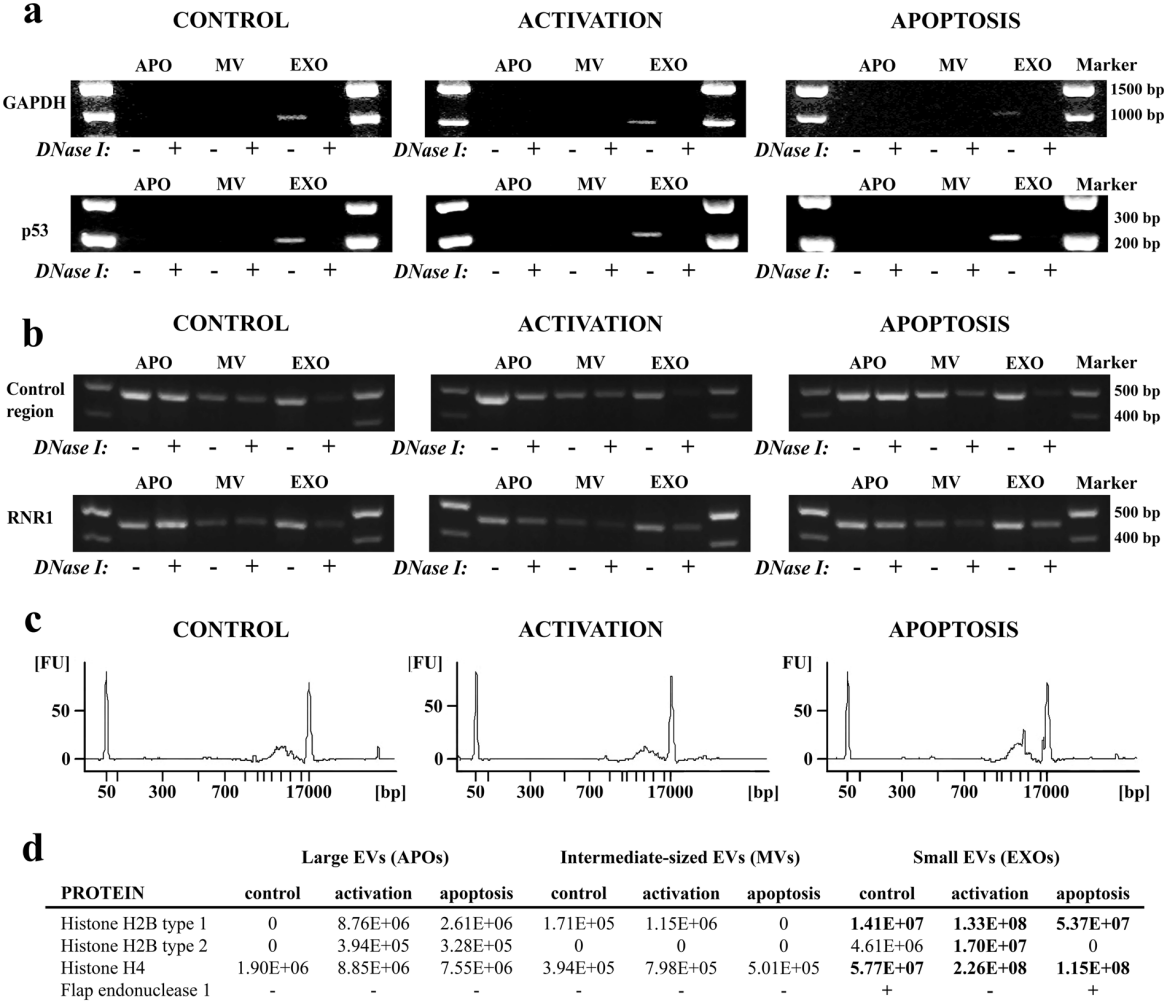
Assessment of DNA and DNA-binding proteins in extracellular vesicle (EV) preparations. **(a-b)** DNA was purified from apoptotic bodies (APOs), microvesicles (MVs) or exosomes (EXOs) released by ciprofloxacin-exposed control, activated or apoptotic Jurkat cells. **(a)** Nuclear (GAPDH, p53) and **(b)** mitochondrial (control region, RNR1) DNA sequences of DNase I-digested/non-digested EVs were amplified by PCR and analyzed by agarose gel electrophoreses. The figure displays cropped gels. Full-length, uncropped gels are shown in Supplementary Figures S11–S13. (c **)** Detection of exosomal DNA derived from ciprofloxacin-exposed control, activated or apoptotic Jurkat cells using an Agilent 2100 Bioanalyzer (DNA 12,000 Kit). The electropherograms show the size distribution of purified exosomal DNA in base pairs (bp) with DNA markers at 50 bp and 17,000 bp. FU: fluorescence units. **(d)** Semi-quantitative mass spectrometry analysis of DNA-binding histones in EV samples. Values in the table are proportional to the amount of histones found in EVs. The presence of f lap endonuclease 1 (FEN1, also known as a mitochondrial DNA-binding nucleoid protein) was also identified by mass spectrometry.

To study the DNA content of EVs secreted by ciprofloxacin-exposed Jurkat cells, certain nuclear and mtDNA sequences were amplified with PCR. Chromosomal DNA amplicons (GAPDH and p53) were present only in EXO samples, and were sensitive to DNase I digestion of EXOs (Fig. 7a). Moreover, we found evidence also for the presence of mtDNA sequences including mitochondrial (mt) control region and mitochondrially encoded 12S RNA (RNR1) in all EV fractions (Fig. 7b), MVs showing the lowest amounts. While mitochondrial amplicons showed variable decrease after DNase I digestion of APOs and MVs, most bands that represented amplified mtDNA of EXOs were sensitive to DNAse I. Full-length, uncropped gels are shown in Supplementary Figures S11-S13. Bioanalyzer profiles also confirmed the presence of DNA in EXO preparations irrespective of the state of the releasing cell with peaks at around 6,000 bp (Fig. 7c).

### Abundance of mitochondrial DNA in the size-based EV fractions

Quantitative real-time PCR was performed in order to quantify mtDNA sequences in comparison to the genomial p53 sequences in the ciprofloxacin-exposed Jurkat cell-derived EV samples. We calculated the ratio of target mt control region and mt RNR1 to the reference p53 genomial DNA sequence. The calculation was based on the real-time PCR amplification efficiencies and C_t_ values as described by Pfaffl^35^. C_t_ values were determined above the threshold at a constant fluorescence level. Our results show that all three EV fractions (APOs, MVs and EXOs) were enriched in mtDNA sequences compared to the genomial p53 DNA content (Supplementary Fig. S14). The enrichment of mtDNA was the highest in the APO fraction (**p < 0.01, one-way ANOVA).

### MS analysis of control, activated and apoptotic Jurkat cells and the secreted EVs

Afterwards, size-based EV subsets released by control, activated and apoptotic cells were characterized by MS (Supplementary Dataset S2–S8). In EXO samples we found numerous proteins detected earlier in EXOs^36^ (such as CD81, syntenin, MHC proteins and heat shock proteins) (Supplementary Dataset S5). Interestingly, histones (H2B1K, H2B1C, H2B2E, H2B1J, H2B2D and H4) were also present in the EXO preparations (Fig. 7d), while APOs and MVs contained lower amounts of histones. This is in line with our flow cytometry results which also confirmed the presence of histone H2B in ciprofloxacin-exposed Jurkat cell-derived EXOs (Fig. 1c) but not in MVs and APOs (Supplementary Fig. S2). Furthermore, our MS analysis deciphered that EXOs secreted by control and apoptotic cells also carried flap endonuclease 1 (FEN1) (Fig. 7d), an mtDNA binding protein representing a part of the mitochondrial nucleoid^37^.

**Figure 8:**
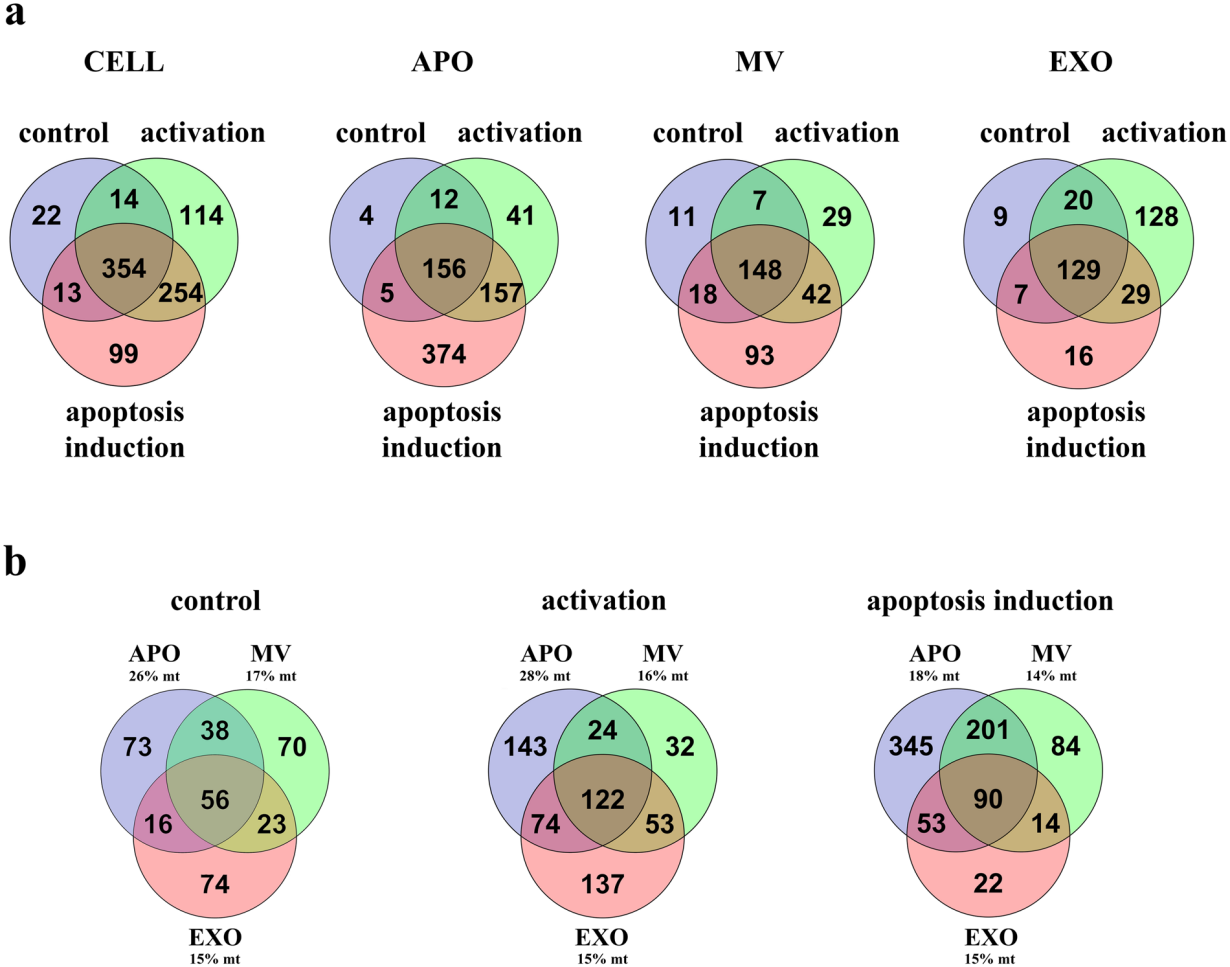
Mass spectrometry analysis of extracellular vesicles (EVs). **(a)** Venn-diagrams of ciprofloxacin-exposed control, activated and apoptotic Jurkat cells and released APOs, MVs and EXOs. **(b)** Venn-diagrams of EVs derived from ciprofloxacin-exposed control, activated and apoptotic Jurkat cells. Percentages of mitochondrial proteins exclusively specific for apoptotic bodies (APOs), microvesicles (MVs) or exosomes (EXOs) are indicated in the graphs, respectively. mt: mitochondrial protein.

Proteomic analysis showed that activated and apoptotic cells expressed high number of functional state-specific proteins (Fig. 8a). APOs and MVs released by apoptotic cells also carried high number of unique proteins (which were not shared with vesicles released by either control or activated cells (Fig. 8a). Apoptotic cell-derived APOs contained among others peroxiredoxin-6, septin 7, septin 9, annexin A7, poly(ADP-ribose) polymerase 1. Proteins unique to apoptotic cell-derived MVs included among others destrin, integrin beta 2, vesicle associated membrane protein 5, tetraspanin-14 and heat shock protein 105 kDa. If we compare EXOs released by control, activated and apoptotic cells, EXOs secreted upon cell activation contained the highest number of unique proteins (Fig. 8a), for example ATP synthase subunit beta, copine-3, replication protein A, cell division control protein, chloride intracellular channel protein 1, tyrosine-protein kinase ITK/TSK.

Gene ontology analysis showed that the highest number of EV-associated proteins with mitochondrial localization was found in APOs (Fig. 8b). In fact, for APOs derived from control and activated cells, 26% and 28% of the APO-specific proteins were associated with the mitochondrial compartment, respectively, which decreased to 18% upon induction of apoptosis (Fig. 8b).

## Discussion

Recently, explosive research interest has focused on cell-derived EVs both as potential biomarkers of various diseases and promising targets or tools for therapy. Except for a recent study^38^, reports on antibiotics-induced cellular responses have mainly focused on cytokines released from antibiotics-exposed cells^39, 40^, and on up-regulated proteins involved in cell cycle arrest^30^ or apoptotic cell death^40, 41^.

In the current work we asked the question whether a sustained (>14 days) exposure to a quinolone antibiotic, ciprofloxacin has an impact on the released EVs of Jurkat cells. Ciprofloxacin has been shown to accumulate intracellularly in leukocytes and affect their cellular functions^39^. Quinolone antibiotics have a concentration-dependent immunomodulatory effect not only in leukocytes (lymphocytes, monocytes), but also on the functions of epithelial and endothelial cells^39^. On the other hand, ciprofloxacin-induced apoptosis of various malignant cells makes ciprofloxacin a candidate anticancer drug^40^.

As a major finding of this study, we found evidence that the presence of ciprofloxacin had an effect on the molecular composition of EVs secreted by Jurkat and MiaPaCa cells. Ciprofloxacin exposure induced the release of substantial amounts of DNA associated with EVs (particularly EXOs). Our experiments demonstrated the abundance of mtDNA associated with different types of Jurkat cell-derived EVs. Unexpectedly, we found that Jurkat EXO-associated mtDNA and also chromosomal DNA were predominantly associated with the external surface of EXOs. Genomic DNA sequences (e.g. GAPDH, p53, KRAS) were previously identified as an internal cargo in EV preparations^14, 20, 42^. Lázaro-Ibánez *et al*. reported the presence of genomic DNA in different size-based EV fractions released by prostate cancer cells^14^. However, the genomic DNA amplicons in the above study showed high variations in different EV fractions, and also depended on the genomic DNA fragment tested. For example, PTEN was not detected in RC92a/hTERT cell-derived EVs upon DNase I digestion similarly to our findings with p53 in Jurkat APOs or MVs. This suggests cell type-specific differences in the EV DNA cargo.

We used for the first time in the EV field a novel resonant waveguide grating-based label-free optical biosensor (Corning® Epic® System)^43–45^ to investigate whether exofacial DNA affected exosomal adhesion properties. Our analysis confirmed that DNAse I sensitive exofacial DNA played a significant role in binding of EXOs to fibronectin. It remains to be established whether DNA by itself or by the associated DNA-binding proteins (also detected on EXOs in this study) were primarily responsible for this interaction, given that exofacial histones were reported earlier to facilitate exosomal adhesion to fibronectin^46^. However, the most exciting question is how DNA is recruited onto the surface of EXOs.

Exposure of Jurkat cells to ciprofloxacin has been shown to induce oxidative stress, production of reactive oxygen species, mitochondrial dysfunction, inhibition of the respiratory chain and decrease of mitochondrial membrane potential leading to mitophagy^47^. Our MS analysis has also confirmed the above biological processes in Jurkat cells. Importantly, the presence of ciprofloxacin has been reported to lead to the loss of mtDNA^28, 29^ and an aneuploidy caused by the genotoxic stress of Jurkat cells^30, 48^. Genotoxic stress response has been shown to induce the release of nucleosomes by leukemic myeloid cells^49^. In the present study, mitochondrial damage of ciprofloxacin-exposed Jurkat cells has been evidenced by the abundance of mtDNA, and the nucleoid protein FEN1, as well as numerous other mitochondrial proteins in the secreted vesicles. Ciprofloxacin inhibits both the bacterial DNA gyrase and the mammalian topoisomerase II enzymes responsible for proper DNA replication^50^. Given that ciprofloxacin mainly inhibits the mitochondrial isoform of mammalian topoisomerase II^29^, its presence induces mtDNA fragmentation as well as subsequent gradual decrease in mtDNA content^29^.

Ciprofloxacin exposure of activated or apoptotic Jurkat cells had no effect on cell viability determined by flow cytometry. However, we detected more histones in EVs derived from activated cells suggesting that ciprofloxacin may accelerate apoptosis of activated Jurkat T cells. Indeed, increased apoptosis induction of activated Jurkat cells by ciprofloxacin has been reported earlier^41^.

Why do antibiotics affect mitochondria of mammalian cells? Mitochondria are of endosymbiotic origin and share numerous features with prokaryotes which may explain their susceptibility to the observed antibiotic-induced damage^27^. Intracellular mitochondrial EV formation and fusion of mitochondrial EVs with multivesicular bodies^51^ may provide a possible mechanism by which mtDNA gets associated with the surface of EXOs. Of note, a strikingly similar mechanism of bacterial DNA release with bacterial outer membrane vesicles (OMVs) has been documented recently^52^. Finally, antibiotic-induced stress response of bacterial cells has been reported to increase the amount of OMV-associated DNA^52^. This OMV-associated DNA also represents a mechanism of horizontal gene transfer of bacteria. However, in mammals, stress-induced release of EXO-associated DNA may rather have an immunomodulatory function. Accumulation of mtDNA in EVs upon oxidative stress induction of human mesenchymal stem cells suppressed inflammatory responses of alveolar macrophages^53^. In contrast, degraded mtDNA has been reported to serve as a danger signal for cells of the innate immunity^54, 55^. Moreover, an antitumor agent topotecan, that inhibits topoisomerase I, induce the secretion of DNA-containing EXOs derived from tumor cells^56^. In the same publication this EXO-associated DNA was shown to elicit inflammation and antitumor immune responses^56^. Binding of DNA-covered EXOs to extracellular matrix proteins (such as fibronectin) may enable antibiotic-induced stressed cells to leave a trail for innate immune cells (similarly to extracellular matrix-bound chemokines).

We found that the exosomal DNA release-inducing effect was not solely observed in the case of Jurkat cells as we also detected ciprofloxacin-induced release of exofacial EV DNA in the case of the pancreatic cancer cell line MiaPaCa. These results demonstrate that DNA-associated EVs may be released from various types of cells after long-term ciprofloxacin exposure.

Our data may suggest a novel EV-related pathway of the removal of damaged mitochondrial and genomial DNA from cells. Given the broad use of antibiotics worldwide, these data warn for possible previously unregarded effects of sustained antibiotic consumption.

## Methods

### Cell cultures, viability

Jurkat human T-cell lymphoma and U937 human histiocytic lymphoma cell lines were purchased from ATCC (Manassas, VA). The MiaPaCa pancreatic cancer cells were kindly provided by Dr Klaus Felix (Universitat Heidelberg, Heidelberg, Germany). Jurkat and U937 cells were cultured in RPMI medium, whereas MiaPaCa cells were grown in DMEM medium, both containing 10% (v/v) fetal bovine serum (FBS), 2 mM glutamine, 0.5% Antibiotic Antimycotic Solution (all from Sigma-Aldrich, St Louis, MO), with or without 10 µg/mL ciprofloxacin (for 14–60 days) at 37 °C in 5% CO_2_/air. The cells were tested regularly for *Mycoplasma* contamination with enzyme immunoassay using *Mycoplasma* Detection Kit (Boehringer Ingelheim, Mannheim, Germany). The viability of Jurkat cells was tested by staining with annexinV-FITC (Sony Biotechnology, San Jose, CE) and propidium iodide (PI, from Sigma) and measured by flow cytometry (using a FACS Calibur flow cytometer, BD Biosciences, San Jose, CA). AnnexinV-labeled cells were diluted 6x with annexinV binding buffer (BD Biosciences, San Jose, CA) to the final volume of 300 µL, and PI was added to the samples in a final concentration of 1 µg/mL before flow cytometry measurements. Results were evaluated using FlowJo software (Treestar, Ashland, OR).

### Activation and apoptosis induction of Jurkat cells

Cells in mid-logarithmic phase were pelleted by centrifugation at 300 g for 10 min, were re-suspended at a density of 8 × 10^5^ cells/mL in serum-free RPMI medium and cultured for 6 or 24 hours. Apoptosis was induced by staurosporine (STS, from Sigma). The optimal STS concentration and incubation time for apoptosis induction were determined by testing the viability (annexinV and propidium iodide (PI) positivity) of cells by flow cytometry. Apoptosis induction was also monitored using the cardiolipin-binding dye nonyl acridin orange (NAO) from Sigma. Cells were stained with NAO at 0.1 µM final concentration in PBS for 20 min at 37 °C, followed by a PBS washing step. Cells were activated by calcium ionophore A23187 in combination with phorbol 12-myristate 13-acetate (PMA) from Sigma. PMA is a known activator of protein kinase C, leading to an increased interleukin-2 transcription (IL-2) in T cells. The combination of PMA and A23187 induces IL-2 mRNA stabilization of T cells^57^ leading to TCR-independent activation. Since exposure to A23187 results in an immediate increase in the intracellular Ca^2+^ level, the calcium indicator Fluo-4 (Thermo Fisher Scientific, Waltham, MA) was used to assess T cell activation. Jurkat cells were washed once in PBS, re-suspended in RPMI at a density of 10^6^ cells/mL, and stained with Fluo-4 in a concentration of 1 µM for 30 min. After incubation, cells were pelleted at 300 g for 10 min and washed two times in PBS. Cells were re-suspended in RPMI and incubated for an additional 30 min. All incubation steps were carried out at 37 °C. Finally, Fluo-4 labeled cells were pelleted again and re-suspended in annexinV binding buffer. Labeled cells were activated with different concentrations of A23187 and the resulting calcium ion influx was monitored in time^58^. For EV isolation and characterization, we selected 0.5 µM STS, and 0.1 µM A23187 in combination with 20 ng/mL PMA for apoptosis induction and cell activation, respectively. Apoptosis and activation of Jurkat cells were carried out in serum-free media, and EVs were isolated from the apoptotic or activated cell supernatant after 6 hours of incubation. The selected STS concentration and incubation time resulted in an increased annexinV positivity of cells with only a limited PI positivity (Supplementary Fig. S7). In the meantime, at the selected concentrations of A23187 and PMA both annexinV and PI stainings were limited (Supplementary Fig. S8). We also confirmed that using the selected A23187 concentration (0.1 µM), a clear Ca^++^ signal was induced in the cells (Supplementary Fig. S9).

Upon ciprofloxacin exposure, control, activated and apoptotic states of cells were also documented using a digital fluorescent microscope (EVOS FL Color Imaging System, Thermo Fisher Scientific). Cells were stained with the green fluorescent membrane dye PKH67 (Sigma) at 2 µM final concentration in Diluent C (Sigma) for 10 min at room temperature, then the staining was stopped by adding the same volume of 10% FBS in RPMI, and the stained cells were pelleted at 300 g for 10 min. PKH67-labeled cells were washed twice in PBS to get rid of the free dye. Cells (5 × 10^5^) were centrifuged onto microscope slides using a cytocentrifuge (Shandon Cytospin3, Thermo Fisher Scientific) and were fixed with 4% paraformaldehyde for 5 min. After washing in PBS, the cover slips were mounted using the Prolong Gold antifade reagent with DAPI (Thermo Fisher Scientific) onto the microscope slides and analyzed using the EVOS FL Color Imaging System.

### EV isolation from conditioned media of cell cultures

For comparison of EVs produced by Jurkat, U937 and MiaPaCa cells in the presence or absence of ciprofloxacin, cells were incubated under serum-free condition for 24 hours. For the comparison of EVs produced by activated or apoptotic Jurkat cells in the presence of ciprofloxacin, cells were cultured for 6 hours under serum- free condition. After the incubation time, size-based EV fractions were isolated including small EVs ~100 nm (EXOs), intermediate sized vesicles in the range of 100–1000 nm (MVs) and large vesicles >1 µm (APOs). A combination of multistep differential centrifugation and hydrostatic filtration was used for EV isolation^3, 4^. Hydrostatic filtration was used throughout this study to avoid fragmentation of EVs by high pressure filtration. Briefly, cells were removed by centrifugation at 300 g for 10 min, and then the supernatant was submitted to a 2,000 g centrifugation for 20 min at 20 °C. The pellet was re-suspended in 4 mL PBS, filtered by gravity through a 5 µm filter (Merck Millipore, Darmstadt, Germany) and pelleted again at 2,000 g to obtain the APO pellet. The supernatant after the first 2,000 g centrifugation was filtered by gravity through a 0.8 µm filter (Whatman CA filter, Sigma) and centrifuged at 12,500 g (JA25.15 rotor) for 40 min, at 16 °C. The pellet was re-suspended in 1.5 mL PBS and washed once by using the same centrifugation settings. The supernatant was discarded after this washing step and the MV pellet was re-suspended in the desired buffer for further experiments. Hydrostatic filtration of the supernatant after the first 12,500 g centrifugation step was carried out through a 0.2 µm filter (Minisart CA filter, Sartorius, Goettingen, Germany), and then the filtrate was pelleted at 100,000 g for 70 min, at 4 °C to obtain the EXO pellet. The supernatant was discarded and the EXO pellet was re-suspended in PBS, and washed at 100,000 g for 70 min, at 4 °C. An Optima MAX-XP bench top ultracentrifuge with MLA-55 rotor (Beckman Coulter Inc., Brea, CA) was used to get the 100,000 g pellet. Before measurements, the isolated EV samples were stored in PBS at 4 °C up to one day.

In order to obtain EXOs of higher purity, Optiprep^TM^ density gradient (Sigma) centrifugation was applied after the first 100,000 g pelleting^33^. Discontinuous Optiprep^TM^ gradient was prepared by layering 40%, 20%, 10% and 5% iodixanol solutions diluted in 0.25 M sucrose, 6 mM EDTA, 60 mM Tris-HCl (pH 7.4) on top of each other^33^. Isolated EXOs from 1.7 × 10^8^ cells were overlaid onto the top of the gradient and pelleted at 100,000 g for 18 hours with a MLS-50 rotor. After ultracentrifugation, 9 individual 0.5 mL fractions were collected manually from the top of the gradient (fraction 1 and 2 were pooled), and each fraction was diluted with PBS and pelleted at 100,000 g for 3 hours. The pellets were then incubated with 4 µm aldehyde/sulfate latex beads (Thermo Fisher Scientific) at room temperature for 40 min, followed by blocking with 100 mM glycine for 30 min, and with 1% (w/v) bovine serum albumin (BSA) for 2 hours^4^. After blocking, the samples were diluted with PBS up to 1.5 mL and pelleted at 2,000 g for 15 min and stained for flow cytometry. Densities of Optiprep^TM^ fractions were determined by measuring weight and absorbance of known Optiprep^TM^ dilutions (40%, 20%, 10% and 5%) and Optiprep^TM^ density gradient fractions at wavelength of 340 nm^33^.

### Size distribution and concentration of the different EV subsets

Size-based EV fractions released by Jurkat cells of different functional states were submitted to tunable resistive pulse sensing (TRPS) analysis using a qNano instrument (IZON Science, Cambridge, MA) as described previously^59, 61^. Briefly, serial dilutions were prepared in 0.2 µm filtered PBS from each EV fraction (derived from 30 mL cell supernatant) and measured by qNano. Particle numbers were counted for at least 3 min using 5 mbar pressure and NP100, NP800 and NP2000 nanopore membranes stretched between 45 and 47 mm. Voltage was applied between 0.1 and 0.4 V in order to achieve a stable 120 nA current. Particle size histograms were recorded when root mean square noise was below 12 pA, particle rate in time was linear, and at least 500 events were counted. Calibration was performed using known concentration of beads CPC100B (mode diameter: 110 nm), CPC800D (mode diameter: 740 nm) and CPC1000E (mode diameter: 900 nm) (all from IZON) diluted 1:1,000 in 0.2 µm filtered PBS. Results were evaluated using IZON Control Suite 3.2 software.

### Transmission electron microscopy of EVs

In order to characterize the morphology and size of the different EV fractions, EV pellets were fixed with 4% paraformaldehyde in PBS for at least 60 min at room temperature and analyzed by transmission electron microscopy (TEM). After washing with PBS, the preparations were postfixed in 1% osmium tetroxide (OsO_4_, Taab, Aldermaston, Berks, UK). This was followed by rinsing with distilled water. The pellets were dehydrated in graded ethanol including block staining with 1% uranyl-acetate in 50% ethanol for 30 min, and were embedded in Taab 812 (Taab). An overnight polymerization of samples at 60 °C was followed by sectioning, and the ultrathin sections were analyzed using a Hitachi 7100 electron microscope (Hitachi Ltd., Japan) equipped by Veleta, a 2000 × 2000 MegaPixel side-mounted TEM CCD camera (Olympus).

### Flow cytometry of EVs

EVs were stained with annexinV-FITC, anti-CD63-PE antibody (Sigma, clone MEM-259), anti-H2B-FITC antibody (Merck Millipore) and PI. APOs, MVs and latex-bound EXOs were stained with annexinV in annexinV binding buffer for 30 min. Latex-bound EXOs were also stained with an anti-CD63 antibody for 30 min. Labeled samples were diluted with annexinV binding buffer 6x into a final volume of 300 µL. Non-stained samples were labeled with 1 µg/mL PI directly before the measurements. At least 10,000 events from equal sample volumes were counted for 1 min at slow flow rate. To verify the vesicular nature of MVs and APOs and to exclude the presence of protein aggregates, we added Triton X-100 in a 0.1% final concentration to the samples^34, 60^. Instrument settings and gates for APOs and MVs were set using staining controls (stainings in annexinV binding buffer). Gates for latex-bound EXOs were set using bare latex beads processed as EXO-covered beads. Data were analyzed by FlowJo software.

### Analysis of vesicular DNA

DNA content of size-based EV fractions was analyzed with/without DNase I (Sigma) digestion. EVs released by 5 × 10^7^ Jurkat cells were centrifuged, and were re-suspended in 190 µL reaction buffer (200 mM Tris-HCl, pH 8.3, 20 mM MgCl). Next, 5 µL of 1 unit/µL DNase I was added to the half of the samples, while the other half was supplemented with 5 µL reaction buffer. After 15 min incubation at room temperature, DNase I enzyme was inactivated using 10 µL of 50 mM EDTA, then 900 µL of 1x ProtectRNA RNase Inhibitor (Sigma) was added into both aliquots, supplemented with PBS. Afterwards, the samples were centrifuged at 100,000 g for 70 min, and were submitted to further analysis.

In order to prove the presence of EXOs after DNase I digestion, both undigested and DNase I-digested EXOs were conjugated onto latex beads and stained with annexinV, anti-CD63 and PI for flow cytometry. The localization of EXO-associated DNA was also analyzed without DNase I at increasing NaCl concentrations assuming that a high salt concentration would decrease the electrostatic interactions between the EXO surface and the attached DNA. First EXOs were conjugated onto latex beads, then the latex-bound EXOs were pelleted, and re-suspended in annexinV binding buffer with increasing NaCl concentrations. Afterwards, all latex-bound EXO samples were pelleted again and re-suspended in regular annexinV binding buffer and were stained with annexinV, anti-CD63 and PI for flow cytometry. Purified nuclear DNA (20 µg) from Jurkat cells was also conjugated onto 10 µL latex beads and analyzed as a control using flow cytometry.

For DNA extraction, cells or purified EV pellets were re-suspended in RBC lysis buffer (Geneaid, New Taipei City, Taiwan) and vortexed for 1 min, and then stored at −20 °C. RNase digestion of lysed EVs was applied to remove any RNA in our EV samples. The DNA content was isolated using a Genomic DNA Mini Kit (Geneaid) according to the instructions of the manufacturer. Finally, DNA was eluted in 30 µL elution buffer and stored at −20 °C until analysis. DNA concentration was determined using a NanoDrop 1000 spectrophotometer (Thermo Fisher Scientific). Purified DNA was either evaluated directly using microfluidic chips (12,000 DNA Chip, Bioanalyzer 2100; Agilent Technologies, Santa Clara, CA) or after PCR amplification using agarose gels. PCR analysis was performed in a GeneAmp PCR System 9700 thermal cycler (Thermo Fisher Scientific), using the primers listed in Supplementary Table S2^62–65^. The following conditions were used: initial denaturation at 95 °C for 4 min, followed by 35 cycles of denaturation (95 °C for 30 sec), annealing (1 min), extension (72 °C for 30 sec), and a final extension step lasting 5 min at 72 °C. Annealing temperatures are also listed in Supplementary Table S2. The PCR reaction mixtures contained 1x Green GoTaq Flexi buffer, 0.6 mM MgCl_2_, 0.2 mM dNTP mix, 3 µM of each primer, 1 unit of GoTaq DNA polymerase (all from Promega, Madison, WI), 10 ng of purified DNA and DEPC H_2_O up to 37.2 µL. Amplified PCR products were stored at 4 °C and analyzed by electrophoresis (100 V for 30 min) in 1.5% agarose for mtDNA and GAPDH and 3% agarose for p53. Gels were visualized on a UV transilluminator using a FluorChem 5500 imaging system with a filter for GelRed dye (Biotium, Fremont, CA).

Real-time quantitative PCR analysis (qPCR) was performed in order to determine the ratio of mitochondrial and genomial DNA sequences in the ciprofloxacin-exposed Jurkat cell-derived EV samples, using an ABI 7900HT Fast Real-Time PCR System (Applied Biosystems). All qPCR reactions were carried out in a 10 μL reaction (with 1 µl of purified DNA) using the SensiFAST^TM^ SYBR Hi-ROX Master Mix (Bioline) and 400 nM of each primer. The thermal cycle parameters were as follows: 1 cycle of stabilization at 50 °C, 10 min; 1 cycle of polymerase activation at 95 °C, 5 min; 45 cycles of denaturation at 95 °C, 15 sec, and annealing/extension at 60 °C, 1 min, and dissociation for 15 sec at 95 °C, 15 sec at 60 °C and 15 sec at 95 °C. Standards and samples were analyzed in triplicates and duplicates, respectively. Melting curves and C_t_ values were analyzed with the SDS 2.4 software (Applied Biosystems).

### Mass spectrometry (MS) of EVs

Two biological replicates were prepared for MS analysis. 20 µL of each sample was digested^66^ following extraction of the proteins^67^. Tryptic peptides were desalted using PierceTM C18 spin columns (Thermo Fisher Scientific) and analyzed using a Dionex Ultimate 3000 Nano LC System (Sunnyvale, CA) coupled to a Bruker Maxis II Q-TOF mass spectrometer (Bremen, Germany) with CaptiveSpray nanoBooster ionization source. In case of the first vesicle isolation separation of the peptides was achieved online using a 15 cm Acclaim Pepmap RSLC nano HPLC column (Thermo Fisher Scientific) following trapping on an Acclaim™ PepMap100™ C18 Nano-Trap column (5 µm, 100 Å, 100 µm × 20 mm, Thermo Fisher Scientific). Peptides originating from the second vesicle isolation were separated online using a 25 cm Waters Peptide BEH C18 nanoACQUITY 1.7 µm particle size UPLC column following the same trapping conditions. Data were processed with ProteinScape 3.0 software (Bruker Daltonik GmbH, Bremen, Germany). Protein identification was performed against Swissprot database (2015_08) using Mascot (Matrix Science, London, UK; version Mascot 2.5) and X! Tandem (The GPM, thegpm.org; version 2007.01.01.1) search engines. The following parameters were applied: Homo sapiens taxonomy, trypsin enzyme, 7 ppm peptide mass tolerance, 0.05 Da fragment mass tolerance, 2 missed cleavages. Carbamidomethylation was set as fixed modification, while deamidation (NQ), oxidation (M) and pyro-carbamidomethylation (N-term C) as variable modifications. Scaffold (version Scaffold_3_00_07, Proteome Software Inc., Portland, OR) software was used to validate peptide and protein identifications as previously published^67^. Label-free quantification of histones was performed using MaxQuant software version 1.5.3.30^68^.

### Assessment of surface adhesion of EVs

To analyze the adhesion of EVs to surface-adsorbed biomolecules, we used the Epic BenchTop (BT) system, a highly sensitive resonant waveguide grating based label-free optical biosensor (Corning Inc., Tewksbury, MA). Each well of the biosensor microplate (Corning® Epic® 384 Well Cell Assay Microplate) contained a sensor unit (a nano-grating embedded in a high-refractive index waveguiding film made of Nb_2_O_5_) at its bottom. The biosensor units were simultaneously illuminated with a light source whose wavelength was swept in a 15,000 pm range with a 0.25 pm resolution. At the resonant wavelength (λ) the light was incoupled to the waveguide film and the excited mode’s evanescent field was penetrated into a 150 nm thick layer above the sensor probing the local refractive index. The resonant wavelength was detected with a CMOS camera after its outcoupling from the biosensor units. Refractive index changes in the sensing zone due to the adhesion of EVs caused a shift in the resonant wavelength (Δλ), which is the primary signal output of the Epic BT system^45, 69^.

Before EV adhesion experiments, wells of an Epic biosensor microplate were pre-coated with either fibronectin (FN), bovine serum albumin (BSA) or poly(L-lysine)-*graft*-poly(ethylene glycol) (PLL-*g-*PEG, Susos, Dübendorf, Switzerland). g = 3.7 is the grafting ratio, indicating the number of lysine units per PEG chains^45^. To measure the molecular adsorption with the biosensor, first a stabile baseline was established with 30 µL PBS in the microplate wells. When the biosensor signal change was less than 5 pm in 5 min (typically obtained within 30 min), the measurement was paused. PBS was replaced with either 50 µg/mL FN or 10 mg/mL BSA coating solutions or with pure PBS. Wells were then incubated for 1 hour at 37 °C. Subsequently, when the microplate was cooled down to room temperature, the measurement was resumed to record the adsorption signals. Then, the coating solutions were removed, and wells were rinsed three times with 30 µL PBS. Next, the uncoated areas were blocked by surface-adsorption of 250 µg/mL PLL-*g*-PEG solution for 30 min at 37 °C. Subsequently, excess solution was removed, and wells were rinsed again three times with PBS. Lastly, 20 µL PBS was pipetted into all wells to record a new baseline of the subsequent EV binding experiments.

Adhesion measurements of EVs were carried out in 40 µL PBS after reaching a stabile biosensor signal in the protein-coated wells. To analyze the role of external DNA of EXO samples in the adhesion onto pre-coated surfaces, identical aliquots of ciprofloxacin-exposed Jurkat cell-derived EXO samples were either digested with DNase I for 15 min at 37 °C, or received DNase I reaction buffer only under the same conditions. Subsequently, 20–20 µL EXO samples were added into each well to measure their adhesion to different surfaces. For control measurements some biosensor wells remained without any surface modifications. The refractive index difference due to the DNase I solution was controlled by using DNase I without EVs. EV samples were analyzed in duplicate wells at room temperature.

### Statistical analysis

For data analysis we used GraphPad Prism v.4. Wilcoxon signed rank test or Mann-Whitney U-test were used to compare two groups in case of parameters with normal or non-normal distribution, respectively. For the comparison of more than two groups we used one-way analysis of variance (ANOVA, Friedman-test). P values of less than 0.05 were considered statistically significant (*P < 0.05, **P < 0.01 and ***P < 0.001). Images were edited with the Adobe Photoshop CS5 software.

## Electronic supplementary material

Supplementary Information

Supplementary Dataset S1

Supplementary Dataset S2

Supplementary Dataset S3

Supplementary Dataset S4

Supplementary Dataset S5

Supplementary Dataset S6

Supplementary Dataset S7

Supplementary Dataset S8
