# Supplementary material for: Antibiotic-induced release of small extracellular vesicles (exosomes) with surface-associated DNA
Source: Sci Rep. 2017 Aug 15;7:8202. doi: 10.1038/s41598-017-08392-1 (PMC5557920; doi:10.1038/s41598-017-08392-1)
Supplement: Supplementary file 1 — Supplementary Information [file 41598_2017_8392_MOESM1_ESM.pdf]

SUPPLEMENTARY INFORMATION

Antibiotic-induced release of small extracellular vesicles (exosomes) with surface-associated DNA

Andrea Németh[1], Norbert Orgovan[2], Barbara W Sódar[1], Xabier Osteikoetxea[1], Krisztina Pálóczi[1], Katalin É Szabó-Taylor[1], Krisztina V Vukman[1], Ágnes Kittel[3], Lilla Turiák[4], Zoltán Wiener[1], Sára Tóth[1], László Drahos[4], Károly Vékey[4], Robert Horvath[2] and Edit I Buzás[1]*

[1]Department of Genetics, Cell- and Immunobiology, Semmelweis University, Budapest, 1085, Hungary

[2]Institute of Technical Physics and Materials Science, Hungarian Academy of Sciences, Budapest, 1121, Hungary

[3]Institute of Experimental Medicine, Hungarian Academy of Sciences, Budapest, 1083, Hungary

[4]Research Centre for Natural Sciences, Hungarian Academy of Sciences, Budapest, 1117, Hungary

* corresponding author:

Prof. Edit I Buzás

edit.buzas@gmail.com

Tel: +36-1-2102929

Fax: +36-1-3036968

Supplementary Figure S1.

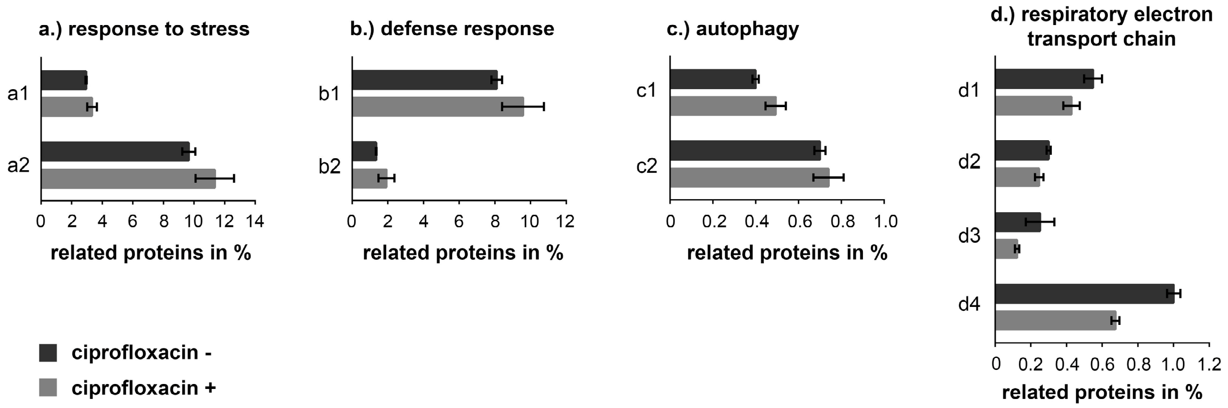

Mass spectrometry analysis of biological processes in Jurkat cells cultured in the absence or sustained presence of ciprofloxacin. (a) Response to stress-, **(b)** defense response-, **(c)** autophagy and **(d)** respiratory electron transport chain-related biological processes of Jurkat cells. Proteins related to the NCBI biological process annotations according to the Scaffold_4.5.1 software. a1: oxidative stress, a2: defense response, b1: innate immune response, b2: inflammatory response, c1: mitochondrion degradation, c2: macroautophagy, d1: mitochondrial electron transport (ubiquitinol to cytochrome c), d2: mitochondrial electron transport (cytochrome c to oxygen), d3: mitochondrial electron transport (NADH to ubiquinone), d4: ATP synthesis coupled electron transport. Data are represented as the mean +/- S.D. (error bars) of two independent experiments.

Supplementary Figure S2.

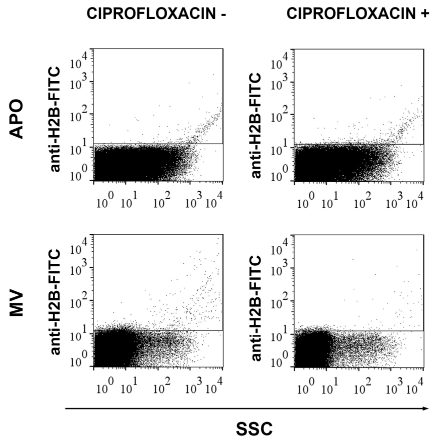

Anti-histone H2B staining of Jurkat cell-derived apoptotic bodies (APOs) and microvesicles (MVs) by flow cytometry. Dot plots show anti-H2B-FITC staining of APOs and MVs derived from Jurkat cells cultured in the absence (-) or presence (+) ciprofloxacin. Black lines indicate the background of anti-H2B-FITC antibody staining in annexin binding buffer.

Supplementary Figure S3.

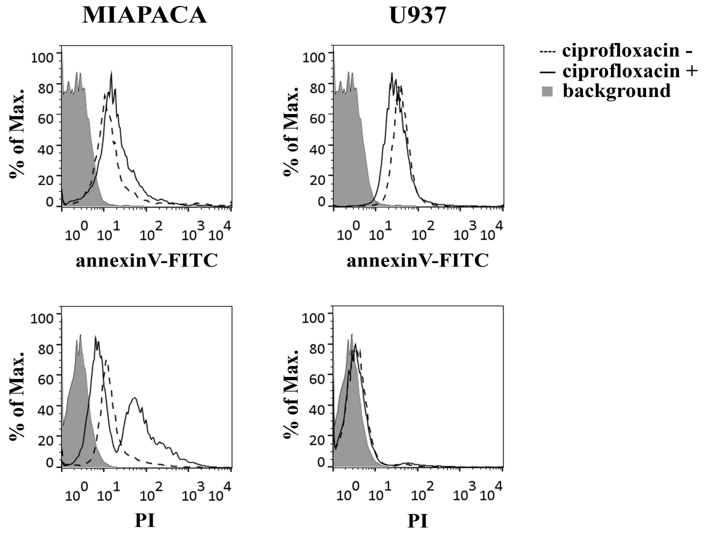

Effect of sustained exposure of MiaPaCa and U937 cells to ciprofloxacin on the composition of the secreted EVs. Exosomes (EXOs)-derived from ciprofloxacin-exposed/unexposed MiaPaCa and U937 cells were conjugated onto latex beads and characterized by flow cytometry after staining with annexinV-FITC and propidium iodide (PI).

The background fluorescence of stained latex beads is indicated by grey histograms. Histograms are representative of two independent experiments.

Supplementary Figure S4.

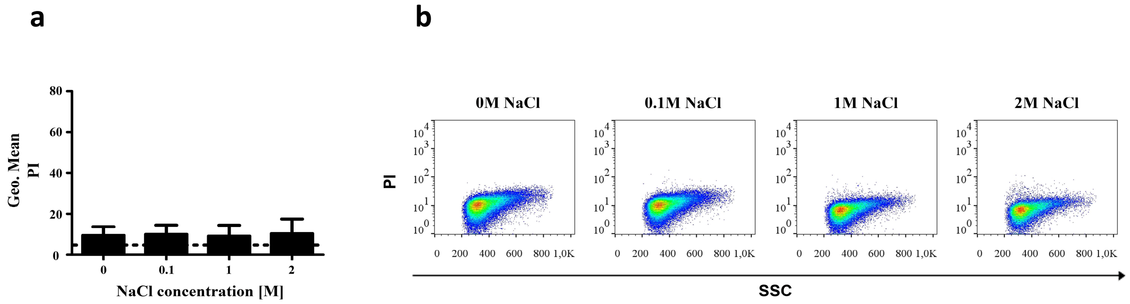

Flow cytometry analysis of latex-bound Jurkat DNA after washing with high salt concentration buffers. (a) Geometric mean values of propidium iodide (PI) fluorescent intensities of latex bead-bound DNA as a function of NaCl washing buffer concentration. Dashed line indicates the PI background of latex-beads without attached DNA. All data are presented as the mean +/- S.D. (error bars) of 3 independent experiments. **(b)** Density plots of PI fluorescent intensity of latex bead-bound DNA as a function of side scatter (SSC) parameter.

Supplementary Figure S5.

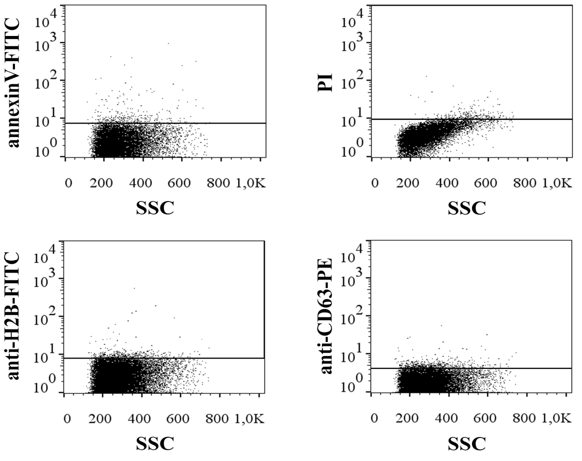

Flow cytometry of labeled latex beads without attached EXOs. Latex beads were labeled with annexinV-FITC, propidium-iodide (PI), anti-H2B-FITC and anti-CD63-PE in order to determine the background of the stainings. Fluorescence intensities are shown as a function of the side scatter (SSC) parameter. Black lines indicate the threshold for positive events.

Supplementary Figure S6.

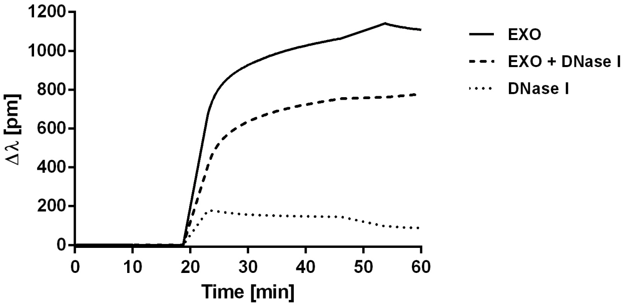

Contribution of DNase I to the adsorption of DNase I-digested exosomes (EXOs). Adsorption curves of undigested EXOs, EXOs digested with DNase I and DNase I only (as a control) represented by continuous, dashed and dotted lines, respectively. After PBS equilibration (20 min), adsorption of DNase I-undigested and digested EXOs as well as DNase I onto bare well Nb_2O_5 surfaces was recorded by measuring the resonance wavelength shift (Δλ) in time.

Supplementary Figure S7.

a b

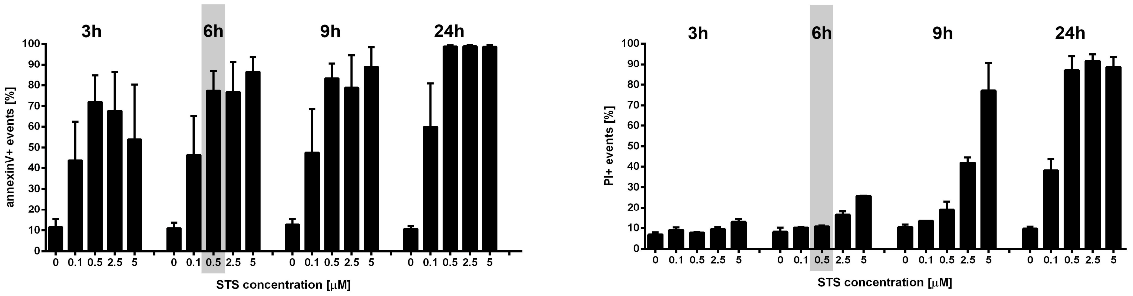

Flow cytometry analysis of the viability of ciprofloxacin-exposed apoptotic Jurkat cells. (a) AnnexinV-FITC and **(b)** propidium iodide (PI) positive events are indicated as a function of staurosporine (STS) concentration and incubation time. Apoptosis was induced in Jurkat cells with different STS concentrations (0.1 µM, 0.5 µM, 2.5 µM and 5 µM) for different periods of time (3h, 6h, 9h and 24h). For further experiments we selected to use 0.5 µM STS for 6 hours (highlighted by grey rectangles) which resulted in a robust increase in annexinV positivity with low PI positive events. All data are presented as the mean +/- S.D. (error bars) of 3 independent experiments.

Supplementary Figure S8.

a b

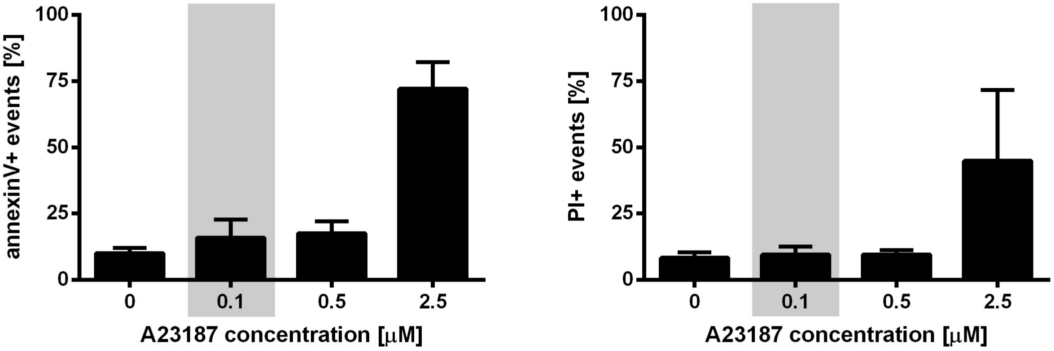

Flow cytometry analysis of the viability of ciprofloxacin-exposed activated Jurkat cells. (a) AnnexinV-FITC and **(b)** propidium iodide (PI) positive events are indicated as a function of Ca^{2+} ionophore A23187 concentration in the presence of 20 ng/ml phorbol 12-myristate 13-acetate after 6 hours incubation. The grey rectangles highlight the results for the selected

0.1 µM A23187 concentration. All data are presented as the mean +/- S.D. (error bars) of 3 independent experiments.

Supplementary Figure S9.

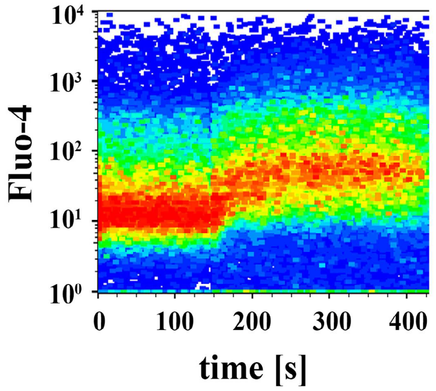

Flow cytometry analysis of Ca^{2+} influx of activated Jurkat cells. Fluo-4 fluorescent intensity of Jurkat cells was recorded as a function of time. After baseline measurement (150 sec), addition of 0.1 µM Ca^{2+} ionophore A23187 resulted in an increase in Fluo-4 signal.

Supplementary Figure S10.

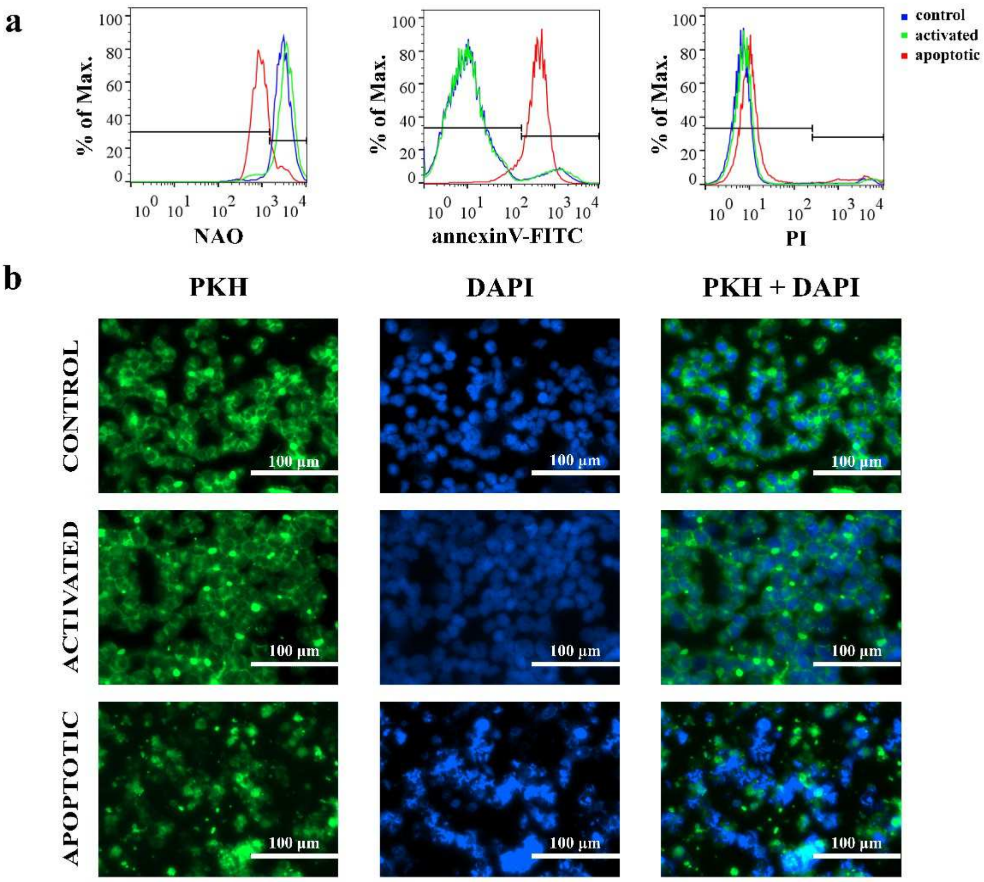

Analysis of ciprofloxacin-exposed control, activated and apoptotic Jurkat cells by flow cytometry and fluorescent microscopy. Activation and apoptosis of Jurkat cells were induced for 6 hours by 0.1 µM A23187 + 20 ng/ml PMA and by 0.5 µM STS, respectively. **(a)** Representative histogram plots of control, activated and apoptotic Jurkat cells analyzed after staining with the cardiolipin-binding dye, nonyl acridin orange (NAO), annexinV-FITC and propidium iodide (PI) for flow cytometry. **(b)** Fluorescent microscopy images of control, activated and apoptotic Jurkat cells after PKH and/or DAPI staining. Activation caused the enlargement of Jurkat cells, which was confirmed by membrane staining with PKH dye. Moreover, DAPI-stained slides show enlarged nuclei of activated cells as compared to control or apoptotic Jurkat cells. Apoptosis resulted in the formation of large apoptotic bodies.

Supplementary Figure S11.

Fig. 7a: GAPDH
CONTROL

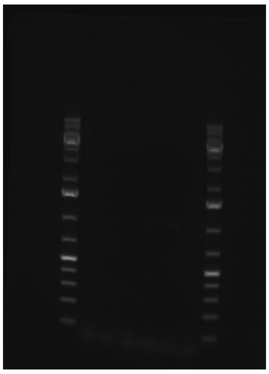

Fig. 7a: GAPDH
ACTIVATION

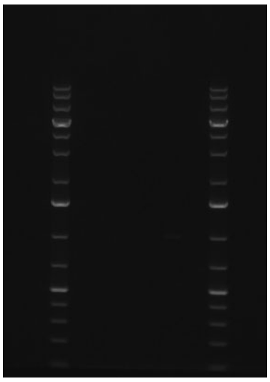

Fig. 7a: GAPDH
APOPTOSIS

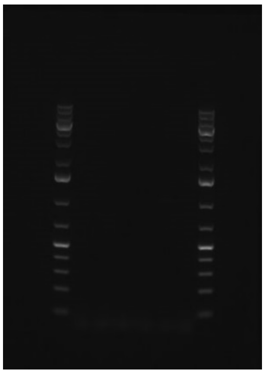

High contrast

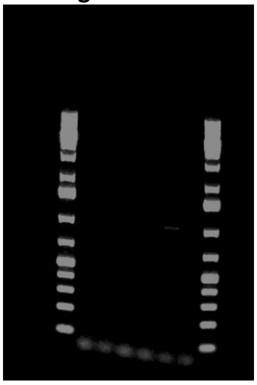

High contrast

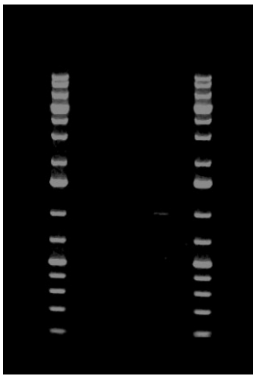

High contrast

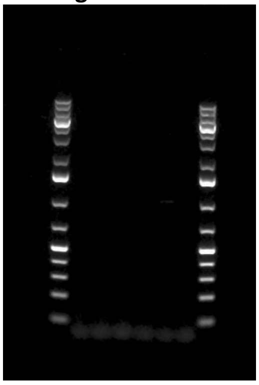

Full-length and high-contrast gel images corresponding to Figure 7a (genomial GAPDH DNA sequence).

Supplementary Figure S12.

Fig. 7a: p53
CONTROL

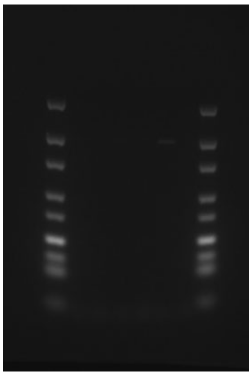

Fig. 7a: p53
ACTIVATION

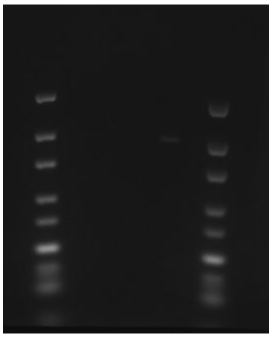

Fig. 7a: p53
APOPTOSIS

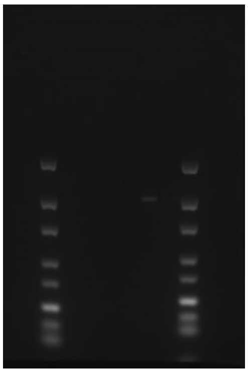

High contrast

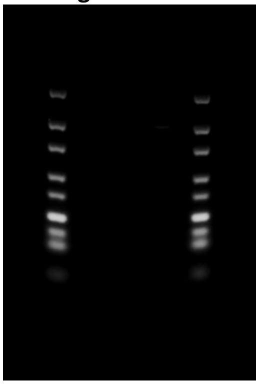

High contrast

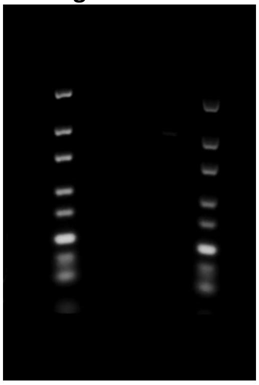

High contrast

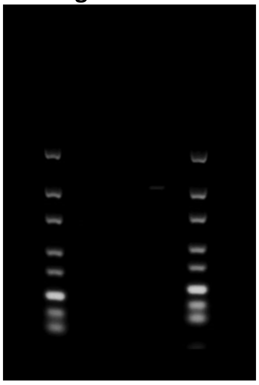

Full-length and high-contrast gel images corresponding to Figure 7a (genomial p53 DNA sequence).

Supplementary Figure S13.

Fig. 7b: mt control region
CONTROL

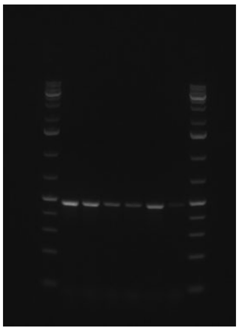

Fig. 7b: mt control region
ACTIVATION

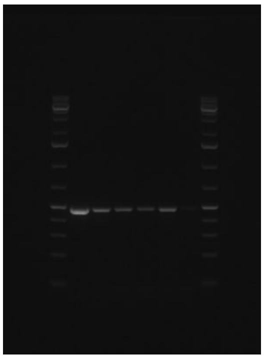

Fig. 7b: mt control region
APOPTOSIS

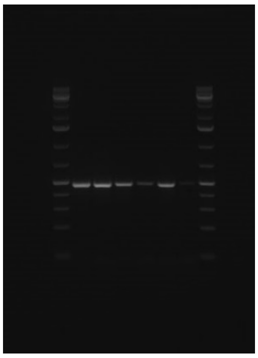

Fig. 7b: mt RNR1
CONTROL

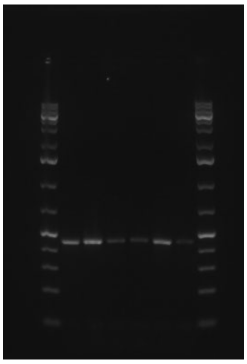

Fig. 7b: mt RNR1
ACTIVATION

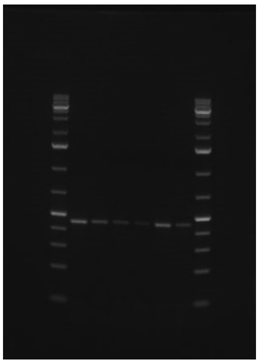

Fig. 7b: mt RNR1
APOPTOSIS

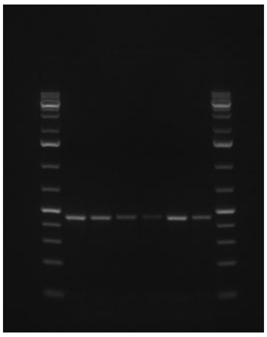

Full-length gel images corresponding to Figure 7b (mt control region and RNR1 DNA sequences).

Supplementary Figure S14.

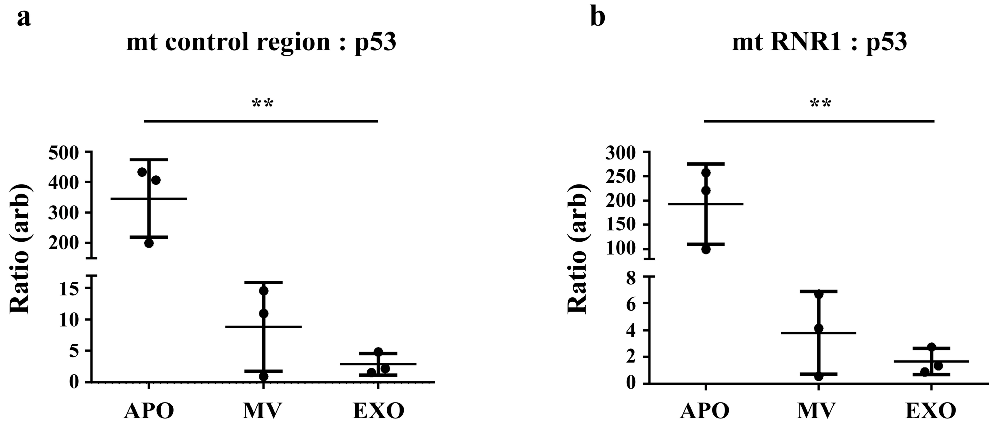

Real time quantitative PCR analysis of ciprofloxacin-exposed Jurkat cell derived apoptotic bodies (APOs), microvesicles (MVs) and exosomes (EXOs). The ratio of target **(a)** mt control region as well as **(b)** mt RNR1 DNA sequence to the reference p53 genomial DNA sequence were calculated. The calculation was based on the real-time PCR amplification efficiencies of target and reference DNA sequences and C_t values. C_t values were determined above a manually obtained threshold at constant fluorescence level. Data are shown as the mean +/- S.D. (error bars) of three independent experiments (**p<0.01, One-way ANOVA). mt: mitochondrial; RNR1: mitochondrially encoded 12S RNA.

Supplementary Table S1.

Fractions	Density (g/mL)
1-2	1.0345
3	1.0449
4	1.0514
5	1.0568
6	1.0719
7	1.0889
8	1.1403
9	1.1946

Densities of Optiprep^{TM} density gradient fractions, containing exosomes highlighted with grey color. Fractions are numbered starting from the top of the gradient.

Supplementary Table S2.

Gene		Sequence 5'-3'	Annealing temperature	Amplicon size (bp)	References
GAPDH	Forward	TGAAGGTCGGAGTCAACGGATTTGGT	65°C	982	[65] Zhang *Et al.*, 2007
	Reverse	CATGTGGGCCATGAGGTCCACCAC			
p53 exon 8	Forward	TCTTGCTTCTCTTTTCCTAT	56°C	188	[62] Hoshida *Et al.*, 2003
	Reverse	CGCTTCTTGTCCTGCTTGCT			
Control region (mitochondrial)	Forward	TTAACTCCACCATTAGCACC	58°C	440	[63] Kjellström *Et al.*, 2012
	Reverse	GAGGATGGTGGTCAAGGGAC			
RNR1 (mitochondrial)	Forward	CAACCTCACCACCTCTTGCT	58°C	470	[64] Li *et al.*, 2015
	Reverse	GTAAGGTGGAGTGGGTTTGG			

Primers used for PCR analysis of nuclear and mitochondrial DNA of extracellular vesicles.
